# The tumor suppressor NDRG2 promotes ACC1 proteasomal degradation and inhibits de novo lipogenesis in hepatocellular carcinoma

**DOI:** 10.1186/s43556-026-00451-2

**Published:** 2026-04-17

**Authors:** Qianqian Shi, Yu Bai, Jiayuan Wang, Mengyao Ru, Kun Zhang, Yan Guo, Zhantao Bai, Lan Shen

**Affiliations:** 1 Department of Biochemistry and Molecular Biology, State Key Laboratory of Cancer Biology, Fourth Military Medical University, Xi’an, 710032 China; 2 State Key Laboratory of Holistic Integrative Management of Gastrointestinal Cancers, Xijing Hospital of Digestive Diseases, Fourth Military Medical University, Xi’an, 710032 China; 3 College of Life Sciences and Research Center for Resource Peptide Drugs, Shaanxi Engineering and Technological Research Center for Conservation and Utilization of Regional Biological Resources, Yan’an University, Yan’an, 716099 China; 4 Department of Oncology, Henan Provincial People’s Hospital, Zhengzhou University People’s Hospital, Zhengzhou, 450003 China

**Keywords:** Hepatocellular carcinoma, Tumor suppressor gene, NDRG2, De novo lipogenesis, ACC1, COP1

## Abstract

Hepatocellular carcinoma (HCC) is a malignant tumor characterized by high metabolic dependence. In particular, it relies on dysregulated lipid synthesis. N-myc downstream regulated gene 2 (NDRG2) acts as a tumor suppressor in HCC, yet its underlying mechanisms remain unclear. This study aimed to elucidate the role of NDRG2 in HCC progression through the regulation of lipid metabolism. We established *NDRG2*-overexpressing and knockout HCC cell lines, and a hepatocellular-specific *Ndrg2*^−/−^ mouse model of induced HCC. Metabolomics, Co-IP, and bioinformatics prediction were used to investigate the regulatory mechanisms and biological functions of NDRG2 on lipogenesis and the activity of the lipogenic enzyme, acetyl-CoA carboxylase 1 (ACC1). Interestingly, compared with wild-type mice, *Ndrg2* knockout mice exhibited significantly enhanced hepatic lipogenesis and hepatocarcinogenesis. Mechanistically, NDRG2 functions as a scaffold protein that specifically recruits the E3 ubiquitin ligase constitutive photomorphogenic protein 1 (COP1), forming an NDRG2-COP1-ACC1 ternary complex, and thereby promoting ACC1 degradation via the ubiquitin–proteasome pathway. Furthermore, the accelerated degradation of ACC1 leads to decreases in de novo lipogenesis (DNL) and lipid droplet formation, thereby reducing the proliferation and progression of HCC cells. Notably, NDRG2-mediated ACC1 degradation significantly synergized with sorafenib to suppress tumor growth and angiogenesis. Our study revealed that NDRG2 mediates the ubiquitination and degradation of ACC1 through recruiting COP1. Thus, targeting the NDRG2-ACC1 axis or its combination with sorafenib may be a novel potential strategy for HCC therapy.

**Supplementary Information:**

The online version contains supplementary material available at 10.1186/s43556-026-00451-2.

## Introduction

Hepatocellular carcinoma (HCC) is the fourth leading cause of cancer-related death worldwide and presents significant therapeutic challenges [1, 2]. Current clinical approaches for treating HCC rely on systemic drug treatments after surgery. In the context of pharmacotherapy, multikinase inhibitors such as sorafenib and lenvatinib serve as first-line treatments. While these treatments can prolong patient survival, their overall therapeutic efficacy remains unsatisfactory [3, 4]. To overcome the limitations of tyrosine kinase inhibitor (TKI) monotherapy, combination strategies have emerged as a pivotal research direction to improve patient outcomes. The core rationale is to combine TKIs with drugs possessing distinct pharmacological mechanisms, leveraging potential synergistic or additive effects to enhance treatment response, overcome resistance and reduce toxicity. Among various approaches, targeting the metabolic reprogramming pathways in HCC has shown considerable promise [5, 6].

HCC is a highly malignant tumor characterized by significant metabolic dysregulation [7, 8], and lipid metabolism reprogramming is one of its hallmark characteristics [9, 10]. In particular, the de novo lipogenesis (DNL) pathway is frequently hyperactivated in HCC. Under normal physiological conditions, hepatocytes primarily rely on exogenous fatty acids from circulation. However, HCC cells exhibit a strong preference for activating DNL to autonomously synthesize large amounts of fatty acids, thereby supporting the high demand for membrane biosynthesis, signaling lipid production, and energy storage required for rapid proliferation [11]. DNL is a tightly regulated multistep enzymatic process involving several key enzymes such as acetyl-CoA carboxylase 1 (ACC1), ATP-citrate lyase (ACLY), fatty acid synthase (FASN), and stearoyl-CoA desaturase 1 (SCD1). In this process, ACC1 catalyzes the carboxylation of acetyl-CoA to malonyl-CoA, which provides the essential substrate for fatty acid chain elongation [12]. ACC1 is a crucial factor in​ HCC prognosis and facilitates​ the malignant phenotypes of HCC through aberrant activation of the​ Wnt/β-catenin signaling pathway. Thus, the heightened activation of DNL is not only a hallmark of lipid metabolic reprogramming in HCC but also a key driver of hepatocarcinogenesis [13–15].

As a tumor suppressor, N-myc downstream regulated gene 2 (NDRG2) plays a pivotal role in the pathogenesis of HCC [16, 17]. NDRG2 not only inhibits the initiation and progression of HCC by regulating the cell cycle and apoptosis, but it also exerts broader effects by interfering with tumor metabolic reprogramming [18–20]. Our previous research revealed that NDRG2 can suppress the aerobic glycolysis and glutaminolysis in tumor cells by inhibiting the c-Myc signaling pathway [21]. In addition, NDRG2 can inhibit pyruvate carboxylase-mediated anaplerosis of the tricarboxylic acid (TCA) cycle, thereby curbing tumor proliferation [22]. Research in renal cell carcinoma further has demonstrated that NDRG2 can synergize with mTOR inhibitors to cooperatively suppress aerobic glycolysis and malignant proliferation, enhancing the efficacy of targeted therapeutics [23]. Our preliminary exploration in lipid metabolism have confirmed that NDRG2 effectively inhibits fatty acid β-oxidation in hepatocytes [24]. Nevertheless, the biological functions and molecular mechanisms through which NDRG2 regulates lipid metabolism in HCC, particularly its impact on the DNL pathway, remain poorly understood [7].

In this study, we investigated the role of NDRG2 in lipid synthesis metabolism and tumorigenesis in HCC. We identified ACC1 as a key downstream target of NDRG2. Our results suggest a novel mechanism by which NDRG2 suppresses hepatocarcinogenesis through the targeting of ACC1-mediated lipid synthesis. We further revealed that NDRG2 regulates the protein stability of ACC1 via the COP1-dependent ubiquitination pathway. Furthermore, we demonstrated a synergistic antitumor effect between NDRG2 and molecular targeted agents such as sorafenib, providing a new theoretical basis for developing combination therapy strategies for HCC based on metabolic intervention.

## Results

### The *Ndrg2* knockout mouse model exhibits increased susceptibility to hepatocellular carcinoma induction and hyperactive hepatic lipogenesis

**Fig. 1:**
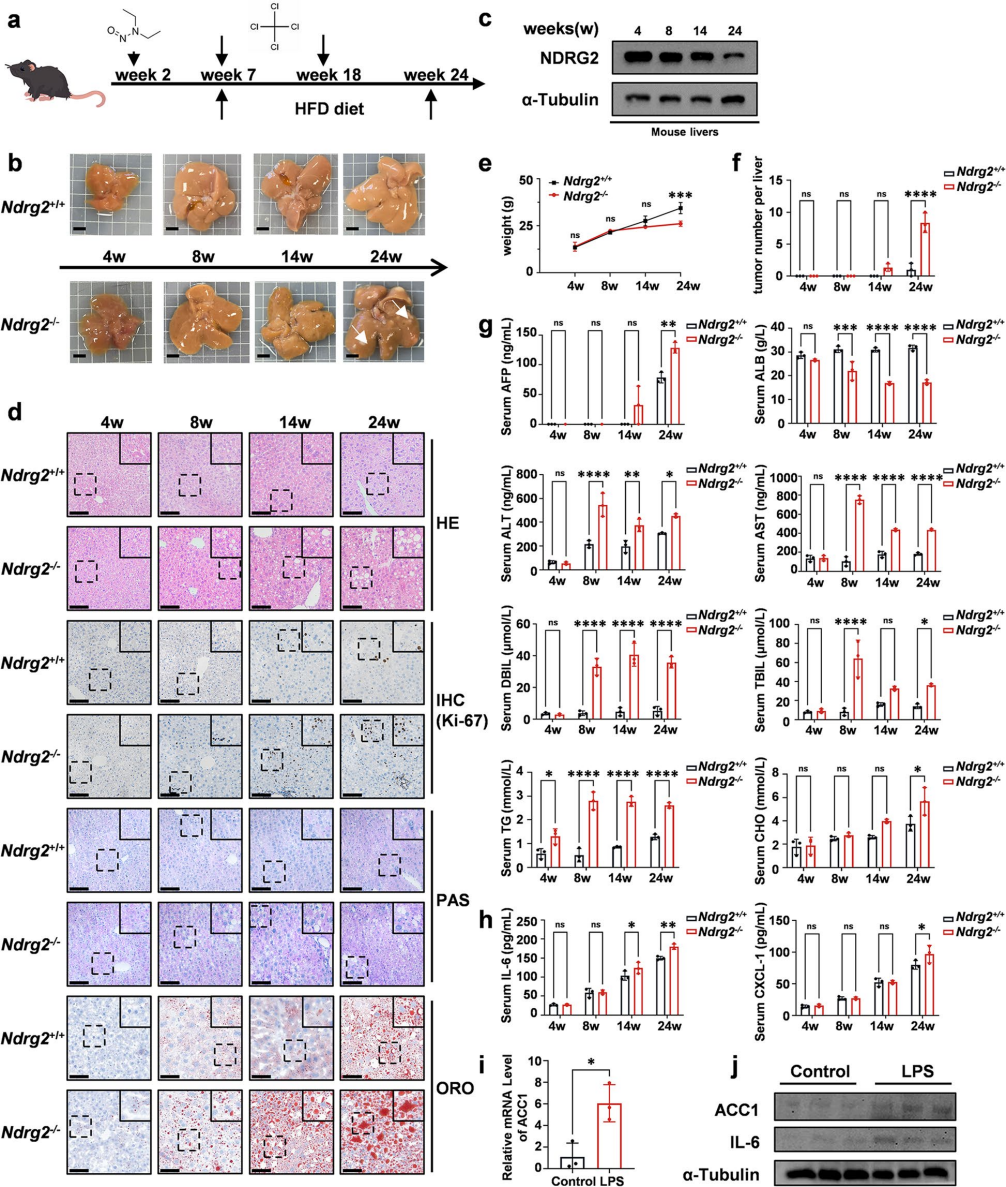
The *Ndrg2* knockout mouse model exhibits increased susceptibility to hepatocellular carcinoma induction and hyperactive hepatic lipogenesis. **a** Schematic diagram of the liver carcinogenesis protocol. This graphic was produced using PowerPoint software. **b** Representative macroscopic images of livers from *Ndrg2*^+*/*+^ and *Ndrg2*^*−/−*^ mice subjected to the liver carcinogenesis protocol at 4, 8, 14, and 24 weeks. Scale bar, 0.5 cm. **c** Western blot analysis of NDRG2 expression in hepatic extracts from *Ndrg2*^+*/*+^ and *Ndrg2*^*−/−*^ mice at the indicated time points.** d** Representative histological analyses of liver tissues, including H&E staining, Ki-67 IHC, PAS staining, and ORO staining. Scale bar, 100 μm. **e** Quantification of body weight in *Ndrg2*^+*/*+^ and *Ndrg2*^*−/−*^ mice (*n* = 6 per group).** f** Tumor number per liver of *Ndrg2*^+*/*+^ and *Ndrg2*.^*−/−*^ mice (*n* = 3 per group).** g** Serum biochemical profiles, including AFP, ALB, ALT, AST, DBIL, TBIL, TG, and CHO levels (*n* = 3 per group).** h** Serum cytokine levels of IL-6 and CXCL-1 (*n* = 3 per group). **i** ACC1 mRNA expression levels in HepG2 cells which were co-cultured with RAW 264.7 cells for 72 h (*n* = 3 per group). The data are presented as the means ± SEMs (error bars) and were compared using two-tailed unpaired t-tests. *P = 0.0161. **j** Western blot analysis of ACC1 and IL-6 expression levels in HepG2 cells which were co-cultured with RAW 264.7 cells for 72 h (*n* = 3 per group). **e–h** The data are presented as the means ± SEMs (error bars) and were compared using the two-way ANOVA; *P < 0.05; **P < 0.01; ***P < 0.001; ****P < 0.0001

To determine the role of NDRG2 in HCC, we first verified that NDRG2 could inhibit the proliferation and activity of tumor cells in two HCC cell lines (Fig. S1a-c). We then established *Ndrg2*^+*/*+^ and *Ndrg2*^*−/−*^ primary liver cancer mouse models (Fig. 1a). During this process, assessments were performed at four time points: 4, 8, 14, and 24 weeks. At these four time points, the incidence and development of tumors were greater in *Ndrg2*^*−/−*^ mice than in *Ndrg2*^+*/*+^ mice (Fig. 1b). In the early stage of tumorigenesis, the level of NDRG2 began to decrease, especially when tumors had already formed at 24 weeks, and the NDRG2 level decreased sharply (Fig. 1c). These finding was consistent with the changes in NDRG2 levels at different stages of HCC observed via bioinformatics analysis (Fig. S2a). We then performed a series of pathological staining experiments.

H&E staining revealed significant differences in liver tissue morphology between the *Ndrg2*^*−/−*^ group and the *Ndrg2*^+*/*+^ control group from 4 to 24 weeks. A comparison of the images at different time points revealed that the arrangement of hepatocytes in the knockout group gradually became disordered with changes in staining intensity in local areas, suggesting potential pathological changes. This difference intensified with increasing age, especially at 14 and 24 weeks, when the destruction of the hepatic cord structure in the knockout group was more obvious. Ki-67 immunohistochemical staining revealed that *Ndrg2* gene knockout significantly enhanced the proliferative activity of mouse liver cells. A comparison of the symmetrical visual fields of the *Ndrg2*^+*/*+^ and *Ndrg2*^*−/−*^ groups revealed that the density of dark brown Ki-67-positive particles in the liver tissue of the *Ndrg2*^*−/−*^ group was significantly greater than that in the *Ndrg2*^+*/*+^ group. The results of PAS and ORO double staining revealed the regulatory effect of *Ndrg2* gene deletion on lipid metabolism in the mouse liver. PAS staining revealed that the extracellular matrix of hepatocytes in the *Ndrg2*^*−/−*^ group significantly presented more dark purple particle deposition, indicating increased glycolipids accumulation, whereas only a light purple background was observed in the *Ndrg2*^+*/*+^ group. ORO staining further confirmed the disordered lipid metabolism in the knockout group, in which the density and area of red lipid droplets in hepatocytes significantly increased, and lipid droplets were fused into sheets in some areas, in sharp contrast to the sparse distribution in the *Ndrg2*^+*/*+^ group (Fig. 1d).

We analyzed the body weights of the mice at four time points: 4, 8, 14, and 24 weeks. In the early stage of tumor development, there was no difference in body weight between the two groups of mice. At 24 weeks, when the tumor was in the development stage, there was a significant difference in body weight between the two groups. The body weight of *Ndrg2*^*−/−*^ mice decreased, whereas that of *Ndrg2*^+*/*+^ mice continued to increase, which was consistent with the development of HCC. We also analyzed the liver/body weight index, and there was no difference in the liver-to-body weight ratio between the two groups of mice (Fig. 1e and S2b). The number of tumors in the liver and the maximal tumor diameter significantly differed between the two groups of mice at 24 weeks (Fig. 1f and S2c). Next, we performed a series of serological analyses of the mice. First, the level of AFP, the most important marker reflecting the occurrence of HCC, significantly differed at 24 weeks, and the same was true for the results of AFP immunohistochemistry (Fig. S2d). In the *Ndrg2*^*−/−*^ group, the levels of ALT and AST significantly increased, indicating hepatocyte damage. Moreover, the levels of DBIL, TBIL, TG and CHO increased, and the level of ALB decreased, revealing multiple phenotypes of cholestasis, lipid metabolism disorders, and synthetic function impairments (Fig. 1g). Additionally, the expression of the inflammatory factors CXCL-1 and IL-6 was increased, further suggesting that *Ndrg2*^*−/−*^ mice were more susceptible to HCC (Fig. 1h). We have also confirmed that sustained elevation of pro-inflammatory cytokines promotes ACC1 expression in HCC, establishing a vicious cycle between chronic inflammation and metabolic dysregulation (Fig. 1i-j and S3a-h).

These results indicate that *Ndrg2* gene knockout promoted the development of HCC and significantly induced the synthesis of glycolipids and lipid droplets.

### NDRG2 suppresses hepatic lipogenesis

**Fig. 2:**
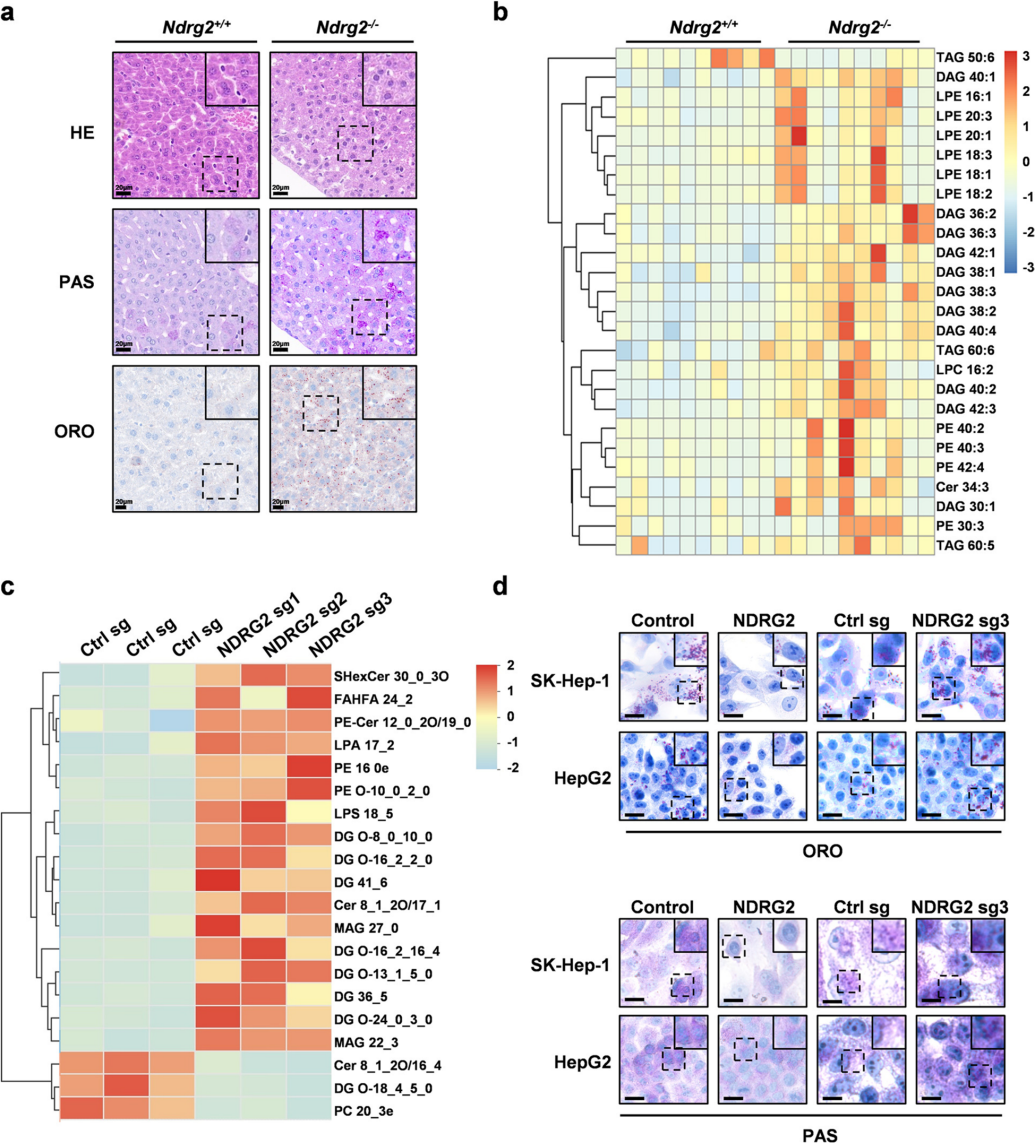
NDRG2 suppresses hepatic lipogenesis. **a** Representative H&E (cell morphology), PAS (glycolipid), and ORO (lipid droplet) staining of liver tissues from *Ndrg2*^+*/*+^ and *Ndrg2*^−/−^ mice. Scale bar, 20 μm.** b** Heatmap of hepatic lipid metabolomics profiles in *Ndrg2*^+/+^ vs. *Ndrg2*^−/−^ mice.** c** Heatmap of hepatic lipid metabolomics profiles in *NDRG2*-knockout vs. control HepG2 cells. **d** ORO (lipid droplets) and PAS (glycolipid) staining in *NDRG2*-overexpressing and *NDRG2*-knockout HCC cells vs. controls. Scale bar, 100 μm

First, we performed H&E, PAS, and ORO staining of untreated *Ndrg2*^+*/*+^ and *Ndrg2*^*-/-*^ mice. H&E staining revealed that the eosin-stained area was more extensive in *Ndrg2*^*-/-*^ mice than in control mice, suggesting better preservation of cytoplasmic components or an increased cell volume, which may be an early sign of steatosis. An analysis of PAS and ORO staining indicated that the presence of the *Ndrg2* gene was likely associated with lipid metabolism, potentially inhibiting the generation of glycolipids and lipid droplets (Fig. 2a). We conducted lipid metabolomic analysis on these two groups of mice (Fig. 2b), the *NDRG2*-knockout HepG2 cell line (Fig. 2c), and the two groups of mice with induced liver cancer models (Fig. S4a-b). The results revealed that, in both the normal and induced groups, the synthesis of phospholipids and triglycerides was significantly increased in *Ndrg2*^-/-^ mice, and the synthesis of phospholipids and diglycerides was significantly increased in the *NDRG2*-knockout cell line. To further validate these findings, we conducted in-depth analyses using ELISA for SPH and DAG (Fig. S4c-f). These results collectively demonstrate that NDRG2 functions as a suppressor of hepatic lipogenesis, both in normal liver tissue and in HCC cells. These finding is consistent with our previous report that NDRG2 overexpression similarly attenuates lipid accumulation in HCC cells [7]. Additionally, PAS and ORO staining of *NDRG2*-overexpressing and *NDRG2*-knockout HepG2 and SK-Hep-1 cells further confirmed that the presence of NDRG2 inhibited the content of glycolipids and lipid droplets in hepatocytes (Fig. 2d).

### NDRG2 physically interacts with ACC1 and exhibits an inverse correlation with clinical specimens

**Fig. 3:**
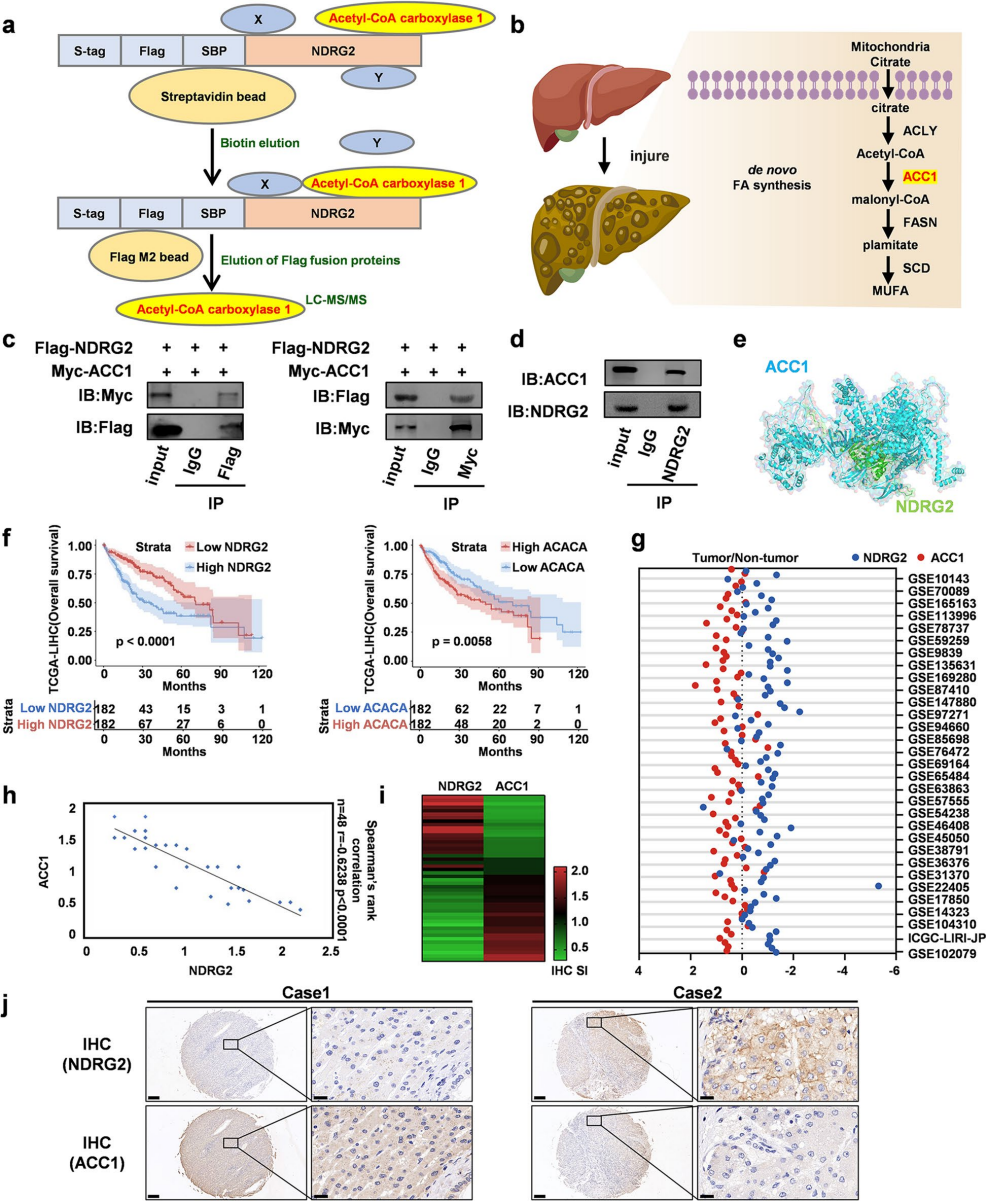
NDRG2 interacts with ACC1, the rate-limiting enzyme of de novo lipogenesis. **a** Graphic illustration of the tandem affinity purification procedure for mass spectrometry (TAP-MS); TAP-MS analysis was used to screen NDRG2-interacting proteins. This graphic was produced using PowerPoint software. **b** Schematic diagram of lipid metabolism in hepatocellular carcinoma. This graphic was produced using PowerPoint software. **c** Co-IP analysis of the NDRG2-ACC1 interaction in 293 T cells.** d** Co-IP analysis of NDRG2-ACC1 interaction in HepG2 cells. **e** Molecular docking simulation of the interaction between NDRG2 and ACC1. **f** TCGA database analysis of the correlation between the expression of NDRG2 (left) and ACC1 (right) and the survival curves of patients with HCC. **g** GEO database quantification of NDRG2 and ACC1 expression in tumor vs. normal tissues. **h** Linear regression analysis of NDRG2 and ACC1 expression levels. **i** Hierarchical clustering of NDRG2 and ACC1 expression profiles. **j** Representative IHC images of tissue microarrays comprising HCC tissues from different differentiation states. Scale bar, 200 μm

To elucidate the molecular mechanism through which NDRG2 suppresses HCC, we performed tandem affinity purification-mass spectrometry (TAP-MS) experiments. The results revealed an interaction between NDRG2 and ACC1, the rate-limiting enzyme in lipid synthesis (Fig. 3a-b). This interaction was confirmed by Co-IP using both exogenously expressed tagged proteins (Fig. 3c) and endogenous proteins in HepG2 cells (Fig. 3d) and was further supported by molecular docking simulations (Fig. 3e).

We next investigated the clinical relevance of the NDRG2-ACC1 interaction. In HCC tissues, the expression level of ACC1 was significantly elevated (Fig. S2a). Survival analysis revealed that high NDRG2 expression was associated with longer patient survival, whereas high ACC1 expression correlated with poorer prognosis (Fig. 3f). Further analysis of multiple GEO datasets for HCC samples revealed that ACC1 expression was higher in tumor tissues, while NDRG2 showed an inverse pattern (Fig. 3g). Correlation analysis between ACC1 and NDRG2 revealed a significant interaction between the two proteins (Fig. 3h-i). In two sets of HCC tissue microarrays, we also observed low expression of NDRG2 and high expression of ACC1 (Fig. 3j). Collectively, these data demonstrate that NDRG2 physically interacts with ACC1 and inversely correlated with ACC1 expression in clinical HCC samples.

### NDRG2 inhibits ACC1 protein content and enzymatic activity

**Fig. 4:**
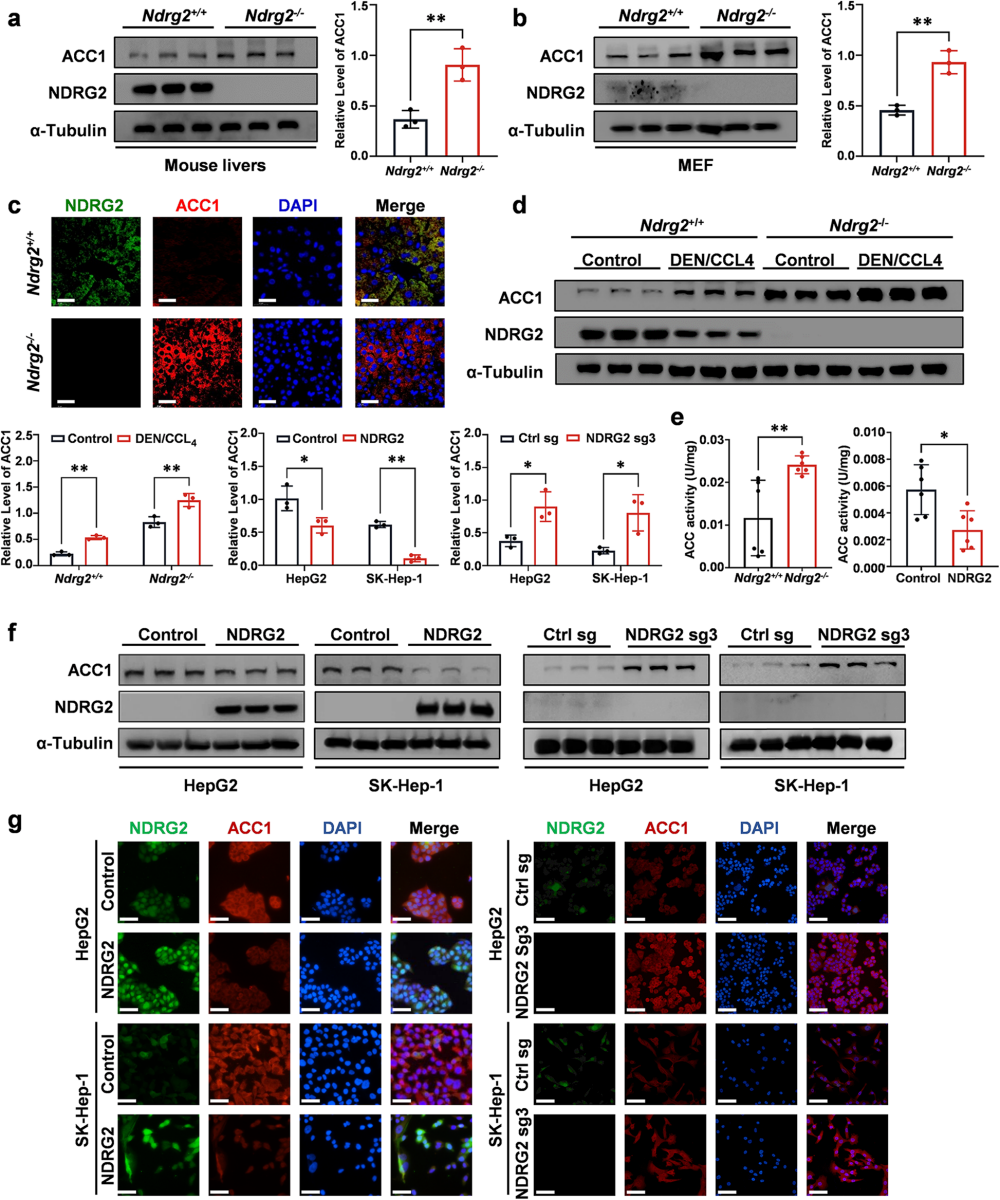
NDRG2 inhibits ACC1 protein content and enzymatic activity.​ **a** Western blot analysis of NDRG2 and ACC1 expression in liver tissues from *Ndrg2*^+/+^ and *Ndrg2*^−/−^ mice. α-Tubulin served as a loading control. **P = 0.0026. **b** Western blot analysis of NDRG2 and ACC1 in MEFs derived from *Ndrg2*^+/+^ and *Ndrg2*^−/−^ mice. The α-Tubulin loading control is shown. **P = 0.0068. **c** Immunofluorescence staining of NDRG2 (green) and ACC1 (red) in liver sections from *Ndrg2*^+/+^ and *Ndrg2*^−/−^ mice. Nuclei were counterstained with DAPI (blue). Scale bar: 50 μm. **d** Western blot analysis of NDRG2 and ACC1 in DEN/CCl_4_-induced HCC tissues from *Ndrg2*^+/+^ and *Ndrg2*^−/−^ mice. **P = 0.0090 (*Ndrg2*^+*/*+^), **P = 0.0015 (*Ndrg2*^*−/−*^). **e** ACC enzymatic activity assays in *Ndrg2*^+/+^ vs. *Ndrg2*^−/−^ mouse livers (left); **P = 0.0072. *NDRG2*-overexpressing vs. control HCC cells (right); *P = 0.0144. **f** Western blot analysis of *NDRG2*-overexpressing vs. control HCC cells (left) and *NDRG2*-knockout vs. control HCC cells (right). *P = 0.0106; **P = 0.0028; *P = 0.0357; *P = 0.0216. **g** Confocal microscopy image showing NDRG2-ACC1 colocalization in HCC cells. Immunofluorescence images were acquired using a Nikon A1R fluorescence microscope. **a-b** (*n* = 3 per group). The data are presented as the means ± SEMs (error bars) and were compared using two-tailed unpaired t-tests. **e** (*n* = 6 per group). The data are presented as the means ± SEMs (error bars) and were compared using two-tailed unpaired t-tests. **d, f** (*n* = 3 per group). The data are presented as the means ± SEMs (error bars) and were compared using two-way ANOVA

To elucidate the regulatory mechanism of NDRG2 on ACC1, we validated its effects at both the transcriptional and protein levels and observed no significant changes in ACC1 mRNA levels (Fig. S5a-b). In liver tissues from *Ndrg2*^+*/*+^ and *Ndrg2*^*−/−*^ mice, NDRG2 significantly suppressed ACC1 protein expression (Fig. 4a). The same phenomenon was observed in MEFs derived from these mice (Fig. 4b). Indirect immunofluorescence assays further confirmed the interaction between NDRG2 and ACC1, revealing a negative correlation between the two proteins (Fig. 4c). In DEN/CCL_4_-induced HCC model mice, the expression of NDRG2 was markedly reduced, whereas the expression of ACC1 was significantly elevated (Fig. 4d). We further assessed ACC1 enzymatic activity and demonstrated that NDRG2 not only inhibited ACC1 protein expression but also suppressed ACC1 enzymatic activity (Fig. 4e). Given that ACC1 activity is also regulated by the AMPK pathway, we examined this axis and found that NDRG2 inhibits AMPK phosphorylation, suggesting the potential existence of an additional regulatory mechanism. (Fig. S5c-e). These findings were validated in HCC cell lines with *NDRG2* overexpression or knockout through Western blot and indirect immunofluorescence assays (Fig. 4f-g). Finally, rescue experiments further confirmed that restoring ACC1 expression partially reversed the inhibition of lipid synthesis caused by NDRG2 overexpression. In summary, these data demonstrate that NDRG2 inhibited lipid synthesis by targeting ACC1 (Fig. S6a-c).

### NDRG2 promotes ACC1 degradation through the ubiquitin‒proteasome pathway mediated by the E3 ligase COP1

**Fig. 5:**
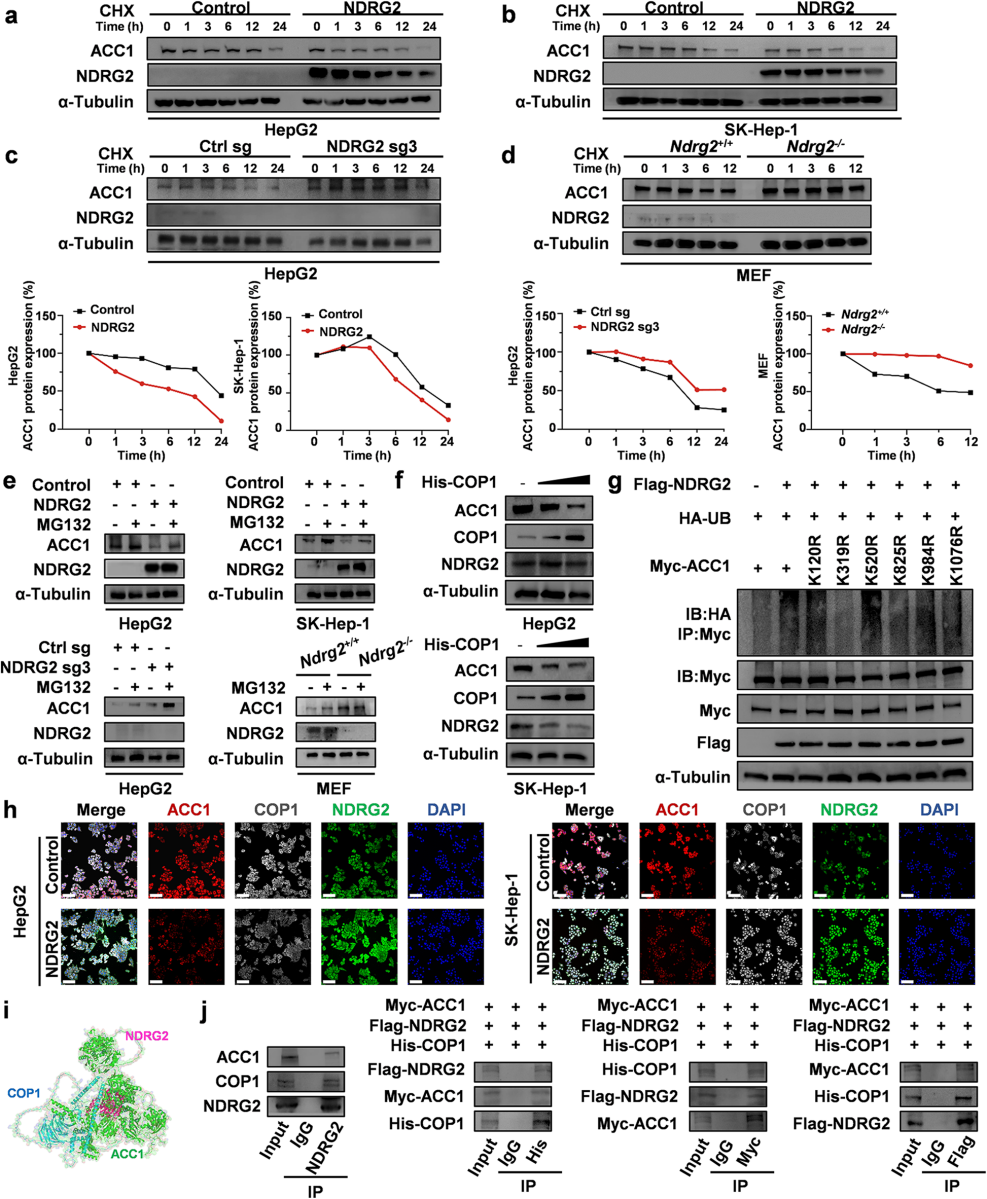
NDRG2 promotes ACC1 degradation through the ubiquitin–proteasome pathway, which is mediated by the E3 ligase COP1. **a-d** Cycloheximide (CHX) chase assays showing ACC1 protein stability in (**a**) *NDRG2*-overexpressing vs. control HepG2 cells (**b**) *NDRG2*-overexpressing vs. control SK-Hep-1 cells (**c**) *NDRG2*-knockout vs. control HepG2 cells (**d**) *Ndrg2*^+/+^ vs. *Ndrg2*^−/−^ MEF cells. Western blots were quantified by normalizing the ACC1 intensity to that of α-tubulin and then to that at the 0-h timepoint.** e** Western blot analysis of NDRG2 and ACC1 in HepG2, SK-Hep-1, and MEF cells treated with 60 μM MG132 for 6 h.** f** Immunoblotting of His-tagged COP1, ACC1, NDRG2 and tubulin proteins in HCC cells co-transfected with different amounts of His-COP1. **g** In vitro ubiquitination assay: Myc-ACC1 (WT, K120R, K319R, K520R, K825R, K984R, and K1076R) immunoprecipitates from 293 T cells co-expressing HA-ubiquitin and Flag-NDRG2 were blotted with the indicated antibodies. **h** Confocal microscopy showing the colocalization of NDRG2 (green), ACC1 (red), and COP1 (white) in HCC cells. Immunofluorescence images were acquired using a Nikon A1R fluorescence microscope. Scale bars: 100 μm. **i** Molecular docking model of the ACC1-NDRG2-COP1 ternary complex. **j** Co-IP validation of the ACC1-NDRG2-COP1 interaction in HepG2 cell lysates and 293 T cell lysates

Since NDRG2 suppresses ACC1 expression only at the protein level, we hypothesized that NDRG2 may regulate ACC1 protein stability or degradation. Cycloheximide (CHX) chase assays in HCC cells and MEFs confirmed that NDRG2 significantly accelerated ACC1 protein degradation (Fig. 5a-d). This degradation was rescued by the proteasome inhibitor MG132 (Fig. 5e), indicating that it is dependent on the ubiquitin–proteasome pathway. We next sought to identify the responsible E3 ubiquitin ligase. Bioinformatic analysis identified COP1, a known regulator of metabolic enzymes (Fig. S7a). Indeed, COP1 overexpression reduced ACC1 levels (Fig. 5f). To map the ubiquitination site, we integrated predictions from the GPS-Uber and UniProt databases (52 potential E3 sites on ACC1) with AlphaFold3-based molecular docking of the ACC1-NDRG2 complex (18 predicted interaction sites). Six candidate lysines at the intersection were mutagenized (K to R) (Fig. S7b-c). Ubiquitination assays revealed K319 as the critical residue for NDRG2-mediated ubiquitination modification of ACC1, as its mutation (K319R) markedly reduced the ubiquitination level of ACC1, while mutations at K120, K520, K984, and K1076 had no effect (Fig. 5g). Immunofluorescence, molecular docking simulations and Co-IP confirmed that NDRG2, COP1 and ACC1 form a ternary complex within cells (Fig. 5h-j, S7d-e). In summary, this study systematically reveals the important role of NDRG2 in the ubiquitination modification of ACC1 and identifies K319 as the key functional site for its ubiquitination-dependent degradation.

### NDRG2 combined with sorafenib predominantly inhibits HCC regression in preclinical models

Given that NDRG2 suppresses HCC by inhibiting the lipogenic enzyme ACC1, we evaluated its therapeutic potential in combination with the first-line agent sorafenib. In vitro NDRG2 overexpression in combination with sorafenib more effectively inhibited HCC cell viability and proliferation than either treatment alone. To further validate the therapeutic relevance of targeting lipogenesis, we included the ACC1 inhibitor TOFA as a positive control, which also showed efficacy (Fig. S8a-c).

**Fig. 6:**
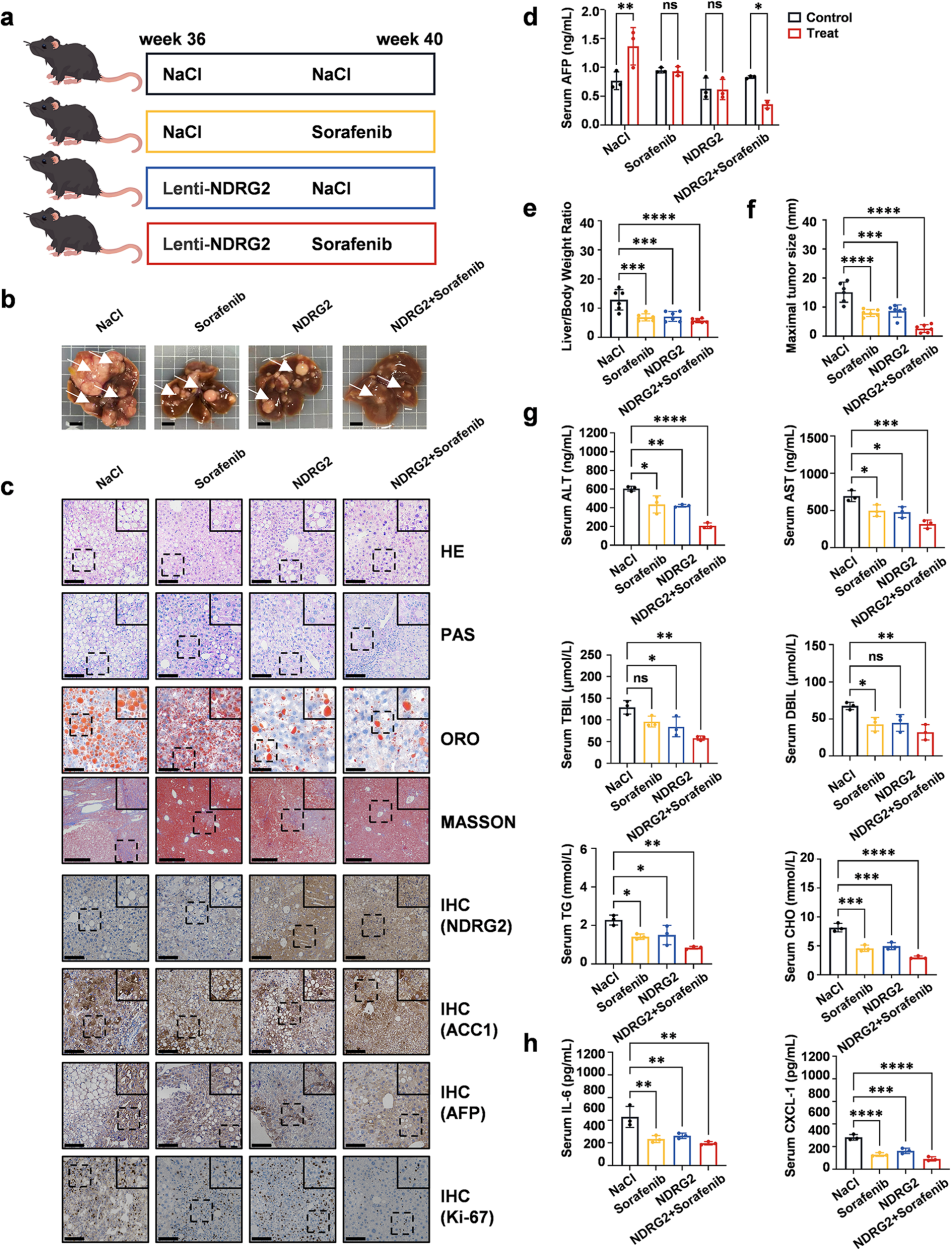
NDRG2 combined with sorafenib predominantly inhibits hepatocellular carcinoma regression in preclinical models. **a** Experimental design of combination therapy in orthotopic HCC. This graphic was produced using PowerPoint software. **b** Representative gross liver morphology of the treatment groups: NaCl, sorafenib monotherapy, NDRG2, and combination therapy. Scale bar, 0.5 cm. **c** Histopathological analysis of HCC tissues showing H&E staining (tumor architecture), PAS (glycogen deposition), ORO (lipid accumulation), Masson (fibrosis), NDRG2 IHC (NDRG2 expression), ACC1 IHC (ACC1 expression), AFP IHC (tumor marker), and Ki-67 IHC (proliferation) results. Scale bars: 100 μm. **d** Serum AFP levels pre- and posttreatment (*n* = 3 per group). The data are presented as the means ± SEMs (error bars) and were compared using two-way ANOVA. **P = 0.0062; *P = 0.0397. **e** Statistical analysis of liver-to-body weight ratio (*n* = 6 per group). **f** Statistical analysis of maximal tumor diameter (*n* = 6 per group). **g** Serum biochemical profiles, including ALT, AST, TBIL, DBIL, TG, and CHO levels (*n* = 3 per group). **h** Serum cytokine levels of IL-6 and CXCL-1 (*n* = 3 per group). **e–h** The data are presented as the means ± SEMs (error bars) and were compared using one-way ANOVA. *P < 0.05, **P < 0.01, ***P < 0.001, ****P < 0.0001

To evaluate its clinical potential, we investigated the therapeutic effects of NDRG2 in combination with the first-line drug sorafenib in both female (Fig. S10a-f) and male mouse models (Fig. 6a) [1, 25]. Consistent with these findings, in vivo mouse experiments revealed that tumor growth was suppressed in both the NDRG2-alone and sorafenib-alone groups, with the most significant inhibition observed in the combination group (Fig. 6b and S10a). This enhanced efficacy was associated with reduced ACC1 levels and the modulation of angiogenesis pathways (Fig. S11a).

Analysis of serum AFP levels revealed a sharp increase in the control group after 4 weeks, whereas no significant change was detected in the NDRG2-alone or sorafenib-alone groups, indicating effective tumor suppression. Notably, the combination group exhibited a marked reduction in AFP levels, further confirming the enhanced inhibitory effect on tumor growth. Pathological examinations (H&E, PAS, ORO, Masson, and IHC) of liver and tumor tissues, along with assessments of liver function (AST, ALT, TBIL, DBIL), lipid profiles (TG, CHO) and inflammatory factors (CXCL-1, IL-6), confirmed the superior efficacy of the combined therapy (Fig. 6c-h and S10b-f). Ultrasound imaging further corroborated these findings (Fig. S9a) [26]. These results suggest that targeting lipid synthesis in combination with TKI drugs might represent a promising strategy for treating HCC.

**Fig. 7:**
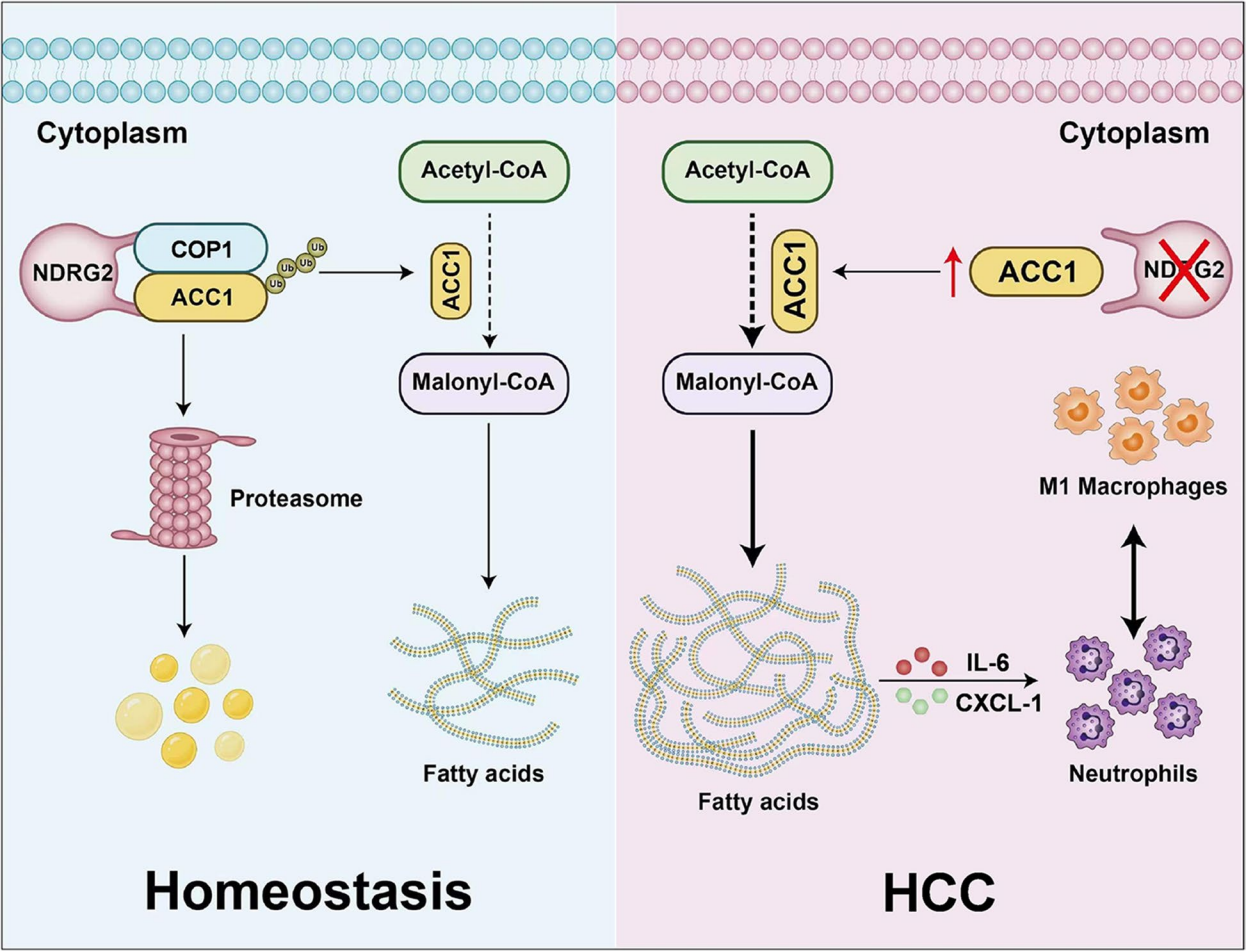
Schematic illustration for the role of NDRG2 in regulating ACC1 and hepatocellular carcinoma tumorigenesis. This diagram contrasts the molecular mechanisms of the NDRG2-COP1-ACC1 protein complex under homeostatic conditions (left, blue background) versus hepatocellular carcinoma (HCC)(right, pink background). In homeostasis, NDRG2 recruits COP1 to mediate the proteasomal degradation of ACC1, thereby limiting malonyl-CoA production and maintaining fatty acid balance. In HCC, NDRG2 deficiency leads to ACC1 accumulation, which drives excessive conversion of acetyl-CoA to malonyl-CoA and promotes aberrant lipogenesis. Additionally, during the prolonged development of HCC, M1 macrophages secrete IL-6 and CXCL1, further promoting ACC1 expression and recruiting neutrophils, establishing a pro-inflammatory and lipogenic vicious cycle. The schematic elucidates the molecular mechanism about NDRG2 loss disrupts ACC1 degradation and drives metabolic dysregulation in HCC. This graphic was produced using Adobe Illustrator software

In summary, during homeostasis, NDRG2 scaffolds COP1 to mediate the ubiquitination and proteasomal degradation of ACC1, thereby limiting malonyl-CoA production and maintaining the fatty acid balance. In HCC, NDRG2 deficiency leads to ACC1 accumulation, which drives excessive conversion of acetyl-CoA to malonyl-CoA, promoting aberrant lipogenesis. Additionally, during the prolonged development of HCC, M1 macrophages secrete IL-6 and CXCL-1, further promoting ACC1 expression and recruiting neutrophils, establishing a vicious proinflammatory and lipogenic cycle (Fig. 7).

## Discussion

DNL exhibits distinct patterns between normal and malignant hepatocytes [27, 28]. While it is quiescent in normal liver cells that primarily utilize dietary lipids, DNL becomes aberrantly activated during hepatocarcinogenesis, serving as a hallmark metabolic feature of HCC. This metabolic pathway involves several rate-limiting enzymes, with ACC1 playing a pivotal role in the first committed step, which involves the catalysis of acetyl-CoA into malonyl-CoA. ACC1 overexpression and hyperactivity in HCC tissues are correlated with tumor aggressiveness, making it a promising therapeutic target among DNL enzymes [29, 30]. The current literature has confirmed that DNL can serve as a therapeutic target for HCC [31]. Previous studies have revealed that the tumor suppressor gene *NDRG2* not only inhibits aerobic glycolysis in HCC [32–34], but also plays a crucial role in regulating DNL in HCC [15, 24]. However, the precise regulatory role of NDRG2 in HCC remains incompletely understood. Our study revealed a potential novel tumor-suppressive mechanism of NDRG2 in HCC, thus demonstrating its role in regulating DNL through inhibition of ACC1.

The activity of ACC1, a classic substrate of AMPK, through phosphorylation. In recent years, increasing attention has been given to the regulation of ACC1 protein abundance, as it participates in adapting to long-term metabolic and functional state changes in cells by modulating protein stability and synthesis rates [35, 36]. Our results demonstrate that NDRG2 can regulate ACC1 protein stability via a unique molecular scaffold mechanism. In the cytoplasm of hepatocytes, NDRG2 specifically recruites the E3 ubiquitin ligase COP1, which has been identified as the E3 ligase for ACC1 [35–37], forming a functional NDRG2-COP1-ACC1 ternary complex that promotes ACC1 ubiquitination and subsequent proteasomal degradation. In HCC cells, the deletion of NDRG2 led to the disruption of ACC1 protein degradation and the hyperactivation of de novo lipogenesis. These findings not only revealed a new tumor-suppressive function of NDRG2 through the inhibition of lipogenesis, but also suggested a potential scaffolding role for NDRG2. Moreover, we also note that NDRG2 may influence AMPK signaling pathways, potentially regulating the phosphorylation modification of ACC1, thereby enabling rapid modulation of ACC1 activity. The coordinated action of these two regulatory mechanisms finely tunes the metabolic function of ACC1 across different time scales and functional levels, working together to maintain cellular lipid metabolic homeostasis. This illustrates the precision and multifunctional adaptability of cellular regulatory networks in coping with complex physiological and pathological conditions.

The malignant phenotype of HCC is characterized not only by metabolic reprogramming but also by the aberrant activation of multiple receptor tyrosine kinase (RTK) signaling pathways [38, 39]. These alterations collectively drive key oncogenic processes, including increased proliferation, increased invasiveness, and pathological angiogenesis [40]. Sorafenib, as the first-generation targeted therapy for treating HCC [41, 42], has demonstrated significant efficacy in advanced patients through the inhibition of RTK signaling pathways [43]. However, the early emergence of drug resistance has substantially limited its long-term therapeutic benefits, which also contributed to the slow progress in HCC targeted drug development during the decade following its approval [44]. Only in recent years have several novel targeted agents been approved for first- or second-line treatment, making the in-depth investigation of sorafenib resistance mechanisms crucial for subsequent drug development [45, 46].

Previous studies have shown that inhibiting lipid synthesis in HCC effectively overcomes resistance to sorafenib [13, 47]. Building upon this foundation, the results of this study demonstrate that NDRG2 can synergize with sorafenib to achieve enhanced therapeutic effects through the regulation of ACC1 and suppressing angiogenesis. Such NDRG2-mediated degradation of ACC1 and subsequent inhibition of fatty acid synthesis work in concert with tyrosine kinase inhibitors to counteract multiple malignant phenotypes of HCC [48, 49].Our experimental evidence confirms that NDRG2 overexpression synergizes with sorafenib to suppress tumor growth, highlighting the promising potential of combining metabolic and targeted therapies for HCC treatment. These findings collectively establish NDRG2 as both a predictive biomarker and a therapeutic target for molecular-guided combination strategies. Although gene therapy has not yet been widely implemented in clinical practice because of various technical, safety, and economic challenges, our study confirms the therapeutic potential of targeting lipid metabolism in combination with sorafenib, offering a new strategic direction for the treatment of HCC.

Our findings provide a new perspective for understanding the complex interplay between inflammation and lipid metabolism. In chronic inflammatory diseases such as HCC, persistent inflammatory factor stimulation may lead to the enhanced expression and accumulation of ACC1, and cause the excessive activation of lipid synthesis. Therefore, there may exist a pro-inflammatory cycle between inflammatory factor stimulation and ACC1-mediated hyperactive lipid synthesis in HCC.

Acute elevation of short-term inflammatory factors may represent an adaptive cellular response to acute stress, aimed at temporarily suppressing energy-consuming lipid synthesis and redirecting resources toward inflammatory responses and cellular repair [50]. However, in chronic inflammatory diseases such as HCC, persistent inflammatory factor stimulation may lead to ACC1 enhancement and accumulation through multiple mechanisms [51]. In HCC treatment, lipid metabolism intervention strategies may need to be differentiated according to disease stage: early stages may require a focus on rapid clearance of inflammatory factors, whereas later stages may necessitate targeted intervention to address abnormal ACC1 stabilization and excessive lipid synthesis activation.

Research on the function of tumor suppressor genes in tumor metabolic reprogramming continues to expand, with significant progress already made in the study of classical tumor suppressors such as TP53 [52, 53]. Our laboratory focused on the NDRG2 gene and reported its multifaceted role in tumor metabolic regulation, including the observation that NDRG2 not only suppresses aerobic glycolysis [23] in tumor cells but also participates in modulating TCA cycle anaplerosis [15] and DNL [7]. These findings provide new perspectives for understanding tumor metabolic reprogramming.

This study has several limitations. Firstly, the complete regulatory network of the tumor suppressor gene NDRG2 remains unclear, particularly the specific mechanisms of its scaffolding function, which require further elucidation. Secondly, the druggability of the NDRG2-COP1-ACC1 axis as a potential therapeutic target still needs to be evaluated. Finally, although gene therapy holds theoretical promise, its clinical application has not been widely implemented due to various challenges in technology, safety, and economics.

In conclusion, our study reveals that NDRG2 functions as a molecular scaffold, specifically recruiting the E3 ubiquitin ligase COP1 to form an NDRG2-COP1-ACC1 ternary complex, which promotes the degradation of ACC1 protein via the ubiquitin–proteasome pathway. The specific degradation of ACC1 induced by NDRG2 inhibits de novo lipogenesis and therefore suppresses the proliferation and progression of hepatocellular carcinoma (HCC) cells. Importantly, NDRG2-mediated metabolic regulation exhibits a significant synergistic anti-tumor effect with molecular targeted agents such as sorafenib. These findings not only reveal a novel tumor-suppressive mechanism of NDRG2 through the inhibition of lipogenesis but also provide a theoretical basis for its role as a predictive biomarker and therapeutic target. This research points to a new strategic direction for combination therapy in HCC, particularly the integration of metabolic intervention and targeted therapy. In the future, in-depth elucidation of the complete regulatory network of NDRG2 and the advancement of its clinical and translational research will lay a solid foundation for developing novel therapeutic strategies of hepatocellular carcinoma.

## Materials and methods

### Study approval

The HCC tissue microarrays used in this study were purchased from the First Affiliated Hospital of Air Force Military Medical University in Xi’an, and written informed consent was obtained from the patients. The use of human samples was approved by the Institutional Ethics Committee of the Air Force Military Medical University. All the animal studies were conducted in accordance with the guidelines provided by the Institutional Animal Care and Use Committee of the Air Force Military Medical University. Sex was considered in the study design and analysis.

### Cell culture

HepG2, SK-Hep-1, RAW264.7 and 293 T cells were purchased from the American Type Culture Collection (Manassas, VA, USA). MEFs were isolated from pregnant *Ndrg2*^+*/*+^ and *Ndrg2*^*−/−*^ mice. All the cells were cultured in Dulbecco’s modified Eagle’s medium (DMEM; Gibco, 6125009) supplemented with 10% fetal bovine serum (FBS; Newzerum, CS500) and 1% penicillin–streptomycin (CellMax, CPS 101.02) at 37 °C with 5% CO₂.

### Reagents and antibodies

Cycloheximide and MG132 were purchased from Selleck; Lipofectamine 2000 was purchased from Thermo Fisher Scientific.

Antibodies against NDRG2 (ab174850, 1:1000 for immunoblotting), PDGFR (ab69506, 1:1000 for immunoblotting), Akt (ab81283, 1:1000 for immunoblotting) and VEGFR (ab11939, 1:1000 for immunoblotting) were purchased from Abcam. Antibodies against NDRG2 (167191–1-Ig, 1:400 for IHC and 1:100 for IP), α-Tubulin (11224–1-AP, 1:1000 for immunoblotting), ACC1 (21923–1-AP, 1:400 for IHC and 1:100 for IP), AMPK (10929–2-AP, 1:1000 for immunoblotting), phospho-ACC1 (29119–1-AP, 1:1000 for immunoblotting), Ki-67 (27309–1-AP, 1:400 for IHC) and IL-6 (21865–1-AP, 1:1000 for immunoblotting) were purchased from Proteintech. Antibodies against ACC1 (#4190, 1:1000 for immunoblotting), phospho-AMPKα (#2535, 1:1000 for immunoblotting), His (#12698, 1:100 for IP) and Myc (#2276, 1:1000 for immunoblotting) were purchased from Cell Signaling Technology. Antibodies against AFP (#41,555, 1:400 for IHC) were purchased from Signalway Antibody, and antibodies against Flag (YM3809), His (YM8314, 1:100 for IP), Myc (YM3002 and YN5506, 1:100 for IP), COP1 (YT1058, 1:1000 for immunoblotting and 1:100 for IP), phospho-VEGFR (YM8555, 1:1000 for immunoblotting) and HA (YM8346, 1:100 for IP and 1:1000 for immunoblotting) were purchased from Immunoway. Antibodies against phospho-Erk1/2 (F0007, 1:1000 for immunoblotting), phospho-PDGFR (F1260, 1:1000 for immunoblotting), Akt (F0004, 1:1000 for immunoblotting) were purchased from Selleck. Antibodies against ERK1/2 (HY-P86312, 1:1000 for immunoblotting) were purchased from MCE.

### Plasmid construction

The Flag-NDRG2 and HA-Ub plasmids were constructed in our laboratory [22]. The Myc-ACC1 (WT, K120R, K319R, K520R, K825R, K984R, and K1076R) and His-COP1 plasmids were constructed by Xi’an GENECARER. An endotoxin-free plasmid extraction kit (DP123-02) was purchased from TianGen. AMP (69–53-4) was purchased from TargetMol.

### Lentiviral transfection

The lentiviruses Lenti-zsgreen-puromycin and Lenti-*NDRG2*-zsgreen-puromycin were developed by Genomeditech. The sgRNA of the *NDRG2* knockout lentivirus was constructed by Gikai Corporation, and the sequences were as follows:

- LV-*NDRG2*-sgRNA1: GATCCTTACCTACCACGATG.
- LV-*NDRG2*-sgRNA2: GTTTCAGTTCGAGGACATGC.
- LV-*NDRG2*-sgRNA3: GAACTTTGTGCGGGTTCATG.

The viruses were added to the HCC cell line for transfection, and puromycin (HepG2: 2 μg/mL; SK-Hep-1: 1 μg/mL) screening was performed for two weeks.

### Tandem affinity purificatio

 293 T cells were transfected with SFB-tagged NDRG2 or empty vector. After 24 h of transfection, the cells were lysed in NETN lysis buffer at 4 °C for 20 min. After centrifugation 13200 rpm for 15 min, the lysate supernatant was collected and incubated with streptavidin-agarose beads at 4 °C overnight. The next day, the beads were washed with NETN wash buffer for five times, followed by NETN elution buffer with 2 mg/mL biotin solution at 4 °C twice. The eluates were then incubated with S-protein agarose beads at 4 °C overnight. After washing the beads with NETN wash buffer for three times, the complexes bound to the S-protein agarose beads were collected, separated by SDS-PAGE, and analyzed by mass spectrometry.

NETN buffer was prepared with 20 mmol/L Tris–HCl (pH 8.0), 100 mmol/L NaCl, 1 mmol/L EDTA, 0.5% NP-40, 50 mmol/L β-glycerophosphate, 10 mmol/L NaF, and 1 mg/mL pepstatin A. Streptavidin-agarose beads were purchased from GE Healthcare Sciences. S-protein agarose beads were purchased from Novagen. Biotin was purchased from Sigma.

### Plate cloning analysis

For the colony formation assay, 1,000 cells per well were seeded in 6-well plates (*n* = 3 per group) with 2 mL of culture medium, and incubated for 10–14 days. The medium was replaced every 4 days, and colony growth was monitored under a microscope. When visible clones formed or the microscopic field became confluent, the medium was removed, the cells were washed with PBS, fixed with 4% paraformaldehyde for 10–20 min, and stained with 0.1% crystal violet for 30 min.

### EdU analysis

Following EdU labeling, cells were incubated for 2 h. The culture medium was discarded, and the cells were washed with PBS, fixed with 4% paraformaldehyde for 15 min, permeabilized with 0.2% Triton X-100 for 10 min, and blocked with 3% BSA for 30 min. EdU and Hoechst staining were performed, and images were subsequently captured under a fluorescence microscope.

### Acetyl-CoA carboxylase activity assay​​

Tissue samples were homogenized in extraction buffer at a 1:5–10 (w/v) ratio, while cell samples were sonicated at a density of 5 × 10^6^ cells/mL. After centrifugation at 8,000 g for 10 min at 4 °C, the supernatant was collected for analysis. The enzymatic reaction was performed in a 200 μL system containing 90 μL of reagent I, 50 μL of reagent II, 40 μL of reagent III, and 10 μL of sample supernatant. Following incubation at 37 °C for 30 min, the reaction was terminated by heating at 90 °C for 5 min. The mixture was then centrifuged at 10,000 g for 5 min, and 20 μL of the supernatant was mixed with 180 μL of freshly prepared phosphomolybdate reagent for color development at 37 °C for 30 min. The absorbance was measured at 660 nm, and the ACC activity (U/mg protein) was calculated as follows: 10 × (A-sample – A-control)/(A-standard – A-blank)/protein concentration. All procedures were performed on ice with freshly prepared reagents. The ACC enzyme activity microassay kit (BC0415) was purchased from Solarbio.

### Immunofluorescence

HCC cell lines with NDRG2 overexpression or knockout were inoculated into 24-well plates, washed with PBS, and fixed with 4% paraformaldehyde. The cells were subsequently incubated with PBS containing 0.2% Triton X-100 and 5% BSA for 1 h, followed by the corresponding primary antibody at 4 °C overnight and the fluorescent secondary antibody at room temperature for 1 h.

A mouse-rabbit-triple-target-four-color-fluorescence-detection-kit was purchased from Immunoway. The goat anti-mouse IgG secondary antibody AF488-conjugated and the goat anti-rabbit IgG secondary antibody AF555-conjugated were purchased from Signalway Antibody.

### Protein half-life assay

To determine the protein half-life of ACC1, cells were treated with Cycloheximide (HepG2: 200 μg/mL; SK-Hep-1: 100 μg/mL; MEF: 100 μg/mL) for the indicated periods (HepG2 and SK-Hep-1: 0, 1, 3, 6, 12, and 24 h; MEF: 0, 1, 3, 6，and 12 h), followed by immunoblotting analysis.

### Immunoblotting

Total protein was extracted from mouse liver tissue samples and HCC cell lines using lysis buffer (Beyotime, P0013B) supplemented with a cocktail (Roche, 04693159001) and phosphatase inhibitors (Roche, 04906837001). The lysates were quantified with BCA reagent, separated by 7.5% SDS-PAGE, transferred to PVDF or NC membranes, and then incubated with primary and secondary antibodies.

### Immunoprecipitation

Mild RIPA buffer containing a protease inhibitor cocktail and phosphatase inhibitors (Beyotime, P1081) was used for cell lysis. The protein lysates were incubated with protein A/G magnetic beads (TargetMol, C0104) conjugated to the primary antibodies at 4 °C for 16 h. The beads were then washed with TBS and analyzed using an immunoblotting protocol.

### Animal studies

A total of 30 *Ndrg2*^+*/*+^ C57BL/6 mice and 90 *Ndrg2*^*−/−*^ C57BL/6 mice were used in this experiment. The mice were housed in a stable environment with a temperature maintained at 24.0 ± 1.0 °C, a humidity of 50.0 ± 5.0%, and a 12-h light–dark cycle. They were housed in cages at a density of 4–6 mice per cage and had free access to food and water. The experimental protocol was approved by the Experimental Animal Welfare and Ethics Committee of the Air Force Military Medical University.

When the mice were 10–14 days old, they were injected with DEN (20 mg/kg, dissolved in PBS) (Selleck, E0136). Starting from the 7th week, the mice in both genotype groups received weekly intraperitoneal injections of CCl₄ (0.5 mL/kg, dissolved in corn oil) [54] (MCE, HY-Y0298) for 12 consecutive weeks. Additionally, they were fed a high-fat diet [55–57].

During the experiment, the mice were sacrificed, and liver tissues were collected at four time points for stage-by-stage analysis: weeks 4, 8, 14, and 24. At week 36, the remaining *Ndrg2*^*−/−*^ mice were randomly divided into 4 groups: the control group, the NDRG2 upregulation group, the sorafenib alone group, and the sorafenib (60 mg/kg/day) (TargetMol, T0093L) plus NDRG2 combination group. These four groups of mice were then treated for 4 weeks.

### Hematoxylin–eosin staining of tissue samples

Paraffin sections were dewaxed and rehydrated in water. The samples were stained with hematoxylin for 2 min, differentiated with 1% HCl-ethanol for 30 s, and stained blue with 0.2% NaHCO₃ and further stained with eosin for 2 min. The samples were immersed in 75%, 85% and 95% ethanol and absolute ethanol I for 3 s each and then in absolute ethanol II, xylene I, and xylene II for 1 min each until clear. The samples were then sealed with neutral gum and examined under a microscope and photographed.

### Periodic Acid-Schiff staining

For tissue paraffin sections, the samples were dewaxed and rehydrated, treated with an oxidizing agent for 5–10 min, rinsed under running water for 5 min, and dried. Schiff’s reagent was added dropwise for 10–15 min of staining, and the samples were washed under running water for 5–10 min and counterstained with Mayer’s hematoxylin for 2 min. Differentiation, bluing, dehydration, clearing, and mounting were then performed.

For the cell climbing experiments, the climbing sections were placed in a 24-well plate, to which the cell suspension was added. After the cells had adhered to the sections, culture medium was added to the wells. The climbing sections were rinsed with PAS reagent, the cells were fixed with 4% paraformaldehyde, and subsequent staining procedures were initiated. The Periodic Acid-Schiff staining kit was purchased from Solarbio.

### Oil Red O staining

For tissue paraffin sections, frozen sections were used. First, the sections were soaked in PBS to remove the OCT embedding medium and fixed with 10% neutral buffered formalin. The sections were subsequently rinsed in distilled water and immersed in 60% isopropanol for 30 s. The prepared Oil Red reagent (Oil Red Solution A:Solution B = 3:2, prepared 10 min in advance) was added for 20 min. The staining reagent was removed, and the sections were washed with 60% isopropanol, stained with hematoxylin for 2 min, differentiated with 1% hydrochloric acid–ethanol for 30 s, stained blue with 0.2% NaHCO₃, rinsed with distilled water, and mounted with glycerin gelatin.

For the cell climbing sections, the operation was the same as that described for the glycogen coverslip staining. The Oil Red O staining kit was purchased from Solarbio.

### Masson staining

The sections were dewaxed, rehydrated, stained with prepared Weigert’s iron hematoxylin solution for 5–10 min, differentiated with 1% hydrochloric acid–ethanol, and washed with water. The sections were then stained blue with Masson bluing solution, washed with water, and then rinsed with distilled water for 1 min. Subsequently, the sections were stained with Ponceau fuchsin solution for 5–10 min, rinsed with weak acid working solution for 1 min, treated with phosphomolybdic acid solution for 1–2 min, and rinsed again with weak acid working solution twice. Finally, the sections were dehydrated, cleared, mounted, and imaged. The Masson staining kit was purchased from Solarbio.

### Enzyme-linked immunosorbent assay

The AFP ELISA kit (FineTest, EM20005), SPH ELISA kit (ELK, 8171), IL-6 ELISA kit (Elascience, E-EL-M0044), CXCL-1 ELISA kit (Elascience, E-EL-M0018) and DAG ELISA kit (SAB, EK4216) were removed from the refrigerator 20 min in advance of the experiment, after which the samples were allowed to equilibrate to room temperature. The serum was thawed and centrifuged again. The washing solution, standards, biotin-antibody working solution, and HRP-streptavidin (SABC) working solution were prepared. The standard wells, sample wells, and blank wells were designated, and their positions were recorded. To minimize experimental errors, triplicate wells were set up for both the standards and the samples. After adding 100 μL of standard or sample per well, the plate was sealed and incubated at 37 °C for 90 min, followed by two washes. Next, 100 μL of biotin‑antibody working solution was added, and the plate was incubated at 37 °C for 60 min before washing three times. Then, 100 μL of SABC working solution was added, and the plate was incubated at 37 °C for 30 min, followed by five washes. Subsequently, 90 μL of TMB substrate was added per well, and the plate was incubated at 37 °C for 20 min in the dark. The reaction was stopped with 50 μL of stop solution per well, and the absorbance at 450 nm was measured immediately for data analysis.

### Co-culture

RAW 264.7 cells were seeded and cultured in good growth condition with 80%–90% confluency. After trypsinization and counting, the cells were resuspended in DMEM complete medium. LPS (Selleck, S7850) was added to a final concentration of 100 ng/mL and mixed thoroughly by pipetting, then the cells were seeded into 6-well plates and incubated at 37 °C in a 5% CO₂ incubator. After 24 h, the cells were washed three times with PBS and incubated in fresh DMEM complete medium. These cells were harvested as M0 macrophages when they reached approximately 80% confluency. Transwell inserts were placed into 6-well plates, then the harvested M0 macrophages were seeded into the upper chamber and HCC cells were seeded into the lower chamber of the 6-well plate. Ensure that both cell types are distributed in their respective compartments. The cells were collected after co-culture for 4 and 72 h.

### Bioinformatic analysis

#### IHGA

The IHGA database (IHGA-Default) query: using the TCGA and GEO databases for survival analysis, gene analysis, and data visualization in HCC. The IHGA database is an interactive web platform designed for HCC research, offering a wealth of gene expression data and analytical tools.

#### UbiBrowser 2.0

The UbiBrowser 2.0 public platform (http://ubibrowser.bio-it.cn/ubibrowser_v2/) was employed to predict potential E3 ubiquitin ligases for ACC1. This platform is a public database specifically designed for predicting ubiquitination regulatory networks.

### Molecular docking simulations

The AlphaFold3 online platform (https://alphafoldserver.com/) was employed for flexible molecular docking simulations. PyMOL was used to create high-quality visualizations of the biomolecular structures.

### Real-time fluorescence quantitative PCR

Total RNA was isolated from cells using an RNA extraction kit (Beyotime, R0026), and complementary DNA (cDNA) was subsequently synthesized via AMV reverse transcriptase (Yeasen, 11141ES60) in accordance with the manufacturer’s instructions. This cDNA was used as a template for quantitative real-time polymerase chain reaction using a SYBR green-based real-time ABI Prism 7500 polymerase chain reaction system (Bio-Rad, Hercules, CA).

### Immunohistochemical staining

The tumors were fixed in 4% paraformaldehyde, embedded in paraffin, and then analyzed by IHC to measure the protein levels of NDRG2, ACC1, AFP and Ki-67. The embedded blocks were obtained via the same method used for H&E staining, and the sections were cut and fixed at 56 °C. The sections were subsequently immersed in PBS to wash away the OCT embedding medium and then dried in an oven at 56 °C. The sections were fixed in 10% neutral buffered formaldehyde solution at room temperature for 10 min. An appropriate amount of endogenous peroxidase blocker was added, followed by incubation at room temperature for 10–20 min and blocking with goat serum for 1 h. Primary antibodies were added, and the sections were incubated at 4 °C overnight. The sections were then rinsed three times with TBS buffer for 5 min each and incubated with the secondary antibody at 37 °C for 1 h. DAB staining was performed, followed by counterstaining with hematoxylin, dehydration, mounting with neutral balsam, and observation and imaging under an optical microscope.

### Statistical analysis

The results are presented as the means ± standard deviations (SDs) of at least three separate experiments for each group. Statistical analysis was performed using ImageJ (USA) and GraphPad Prism (version 8.0, USA) software. For comparisons involving a single variable,​ independent Student's t-tests were used for two-group analyses, while one-way ANOVA​ was utilized for multi-group comparisons. For comparisons involving two independent variables, two-way ANOVA was applied.​

## Supplementary Information

Supplementary Material 1.

## Data Availability

The data and materials that support the findings of this study are available from the corresponding author upon reasonable request.

## Abbreviations

HCC: Hepatocellular carcinoma
DNL: De novo lipogenesis
NDRG2: N-Myc downstream-regulated gene 2
ACC1: Acetyl-CoA carboxylase 1
AFP: Alpha-fetoprotein
AST: Aspartate aminotransferase
ALT: Alanine aminotransferase
TBIL: Total bilirubin
DBIL: Direct bilirubin
TG: Triglyceride
CHO: Cholesterol
SPH: Sphingomyelin
DAG: Diacylglycerol
TAP-MS: Tandem affinity purification-mass spectrometry
LPS: Lipopolysaccharide
MEF: Mouse embryonic fibroblast
COP1: Constitutively photomorphogenic 1
TKI: Tyrosine kinase inhibitor
TCA: Tricarboxylic acid
