# Supplementary material for: The tumor suppressor NDRG2 promotes ACC1 proteasomal degradation and inhibits de novo lipogenesis in hepatocellular carcinoma
Source: Mol Biomed. 2026 Apr 17;7:52. doi: 10.1186/s43556-026-00451-2 (PMC13087075; doi:10.1186/s43556-026-00451-2)
Supplement: Supplementary file 1 — Supplementary Material 1. [file 43556_2026_451_MOESM1_ESM.docx]

**Supplementary Figures and Legends**

**The tumor suppressor NDRG2 promotes ACC1 proteasomal degradation and inhibits de novo lipogenesis in hepatocellular carcinoma**

**Qianqian Shi^1,2,3^, Yu Bai^1,2,3^, Jiayuan Wang^1,2^, Mengyao Ru^1,2^, Kun Zhang^1,2^, Yan Guo^1,2,4^, Zhantao Bai^3^ and Lan Shen^1,2🖂^**

^1^ Department of Biochemistry and Molecular Biology, State Key Laboratory of Cancer Biology, Fourth Military Medical University, Xi'an 710032, China.

^2^ State Key Laboratory of Holistic Integrative Management of Gastrointestinal Cancers, Xijing Hospital of Digestive Diseases, Fourth Military Medical University, Xi'an 710032, China

^3^ College of Life Sciences and Research Center for Resource Peptide Drugs, Shaanxi Engineering and Technological Research Center for Conservation and Utilization of Regional Biological Resources, Yan'an University, Yan'an 716099, China

^4^ Department of Oncology, Henan Provincial People's Hospital, Zhengzhou University People's Hospital, Zhengzhou 450003, China

**🖂 Correspondence**: Lan Shen, Email: [lanshen@fmmu.edu.cn](mailto:lanshen@fmmu.edu.cn). Department of Biochemistry and Molecular Biology, State Key Laboratory of Cancer Biology, Fourth Military Medical University, Xi'an 710032, China.


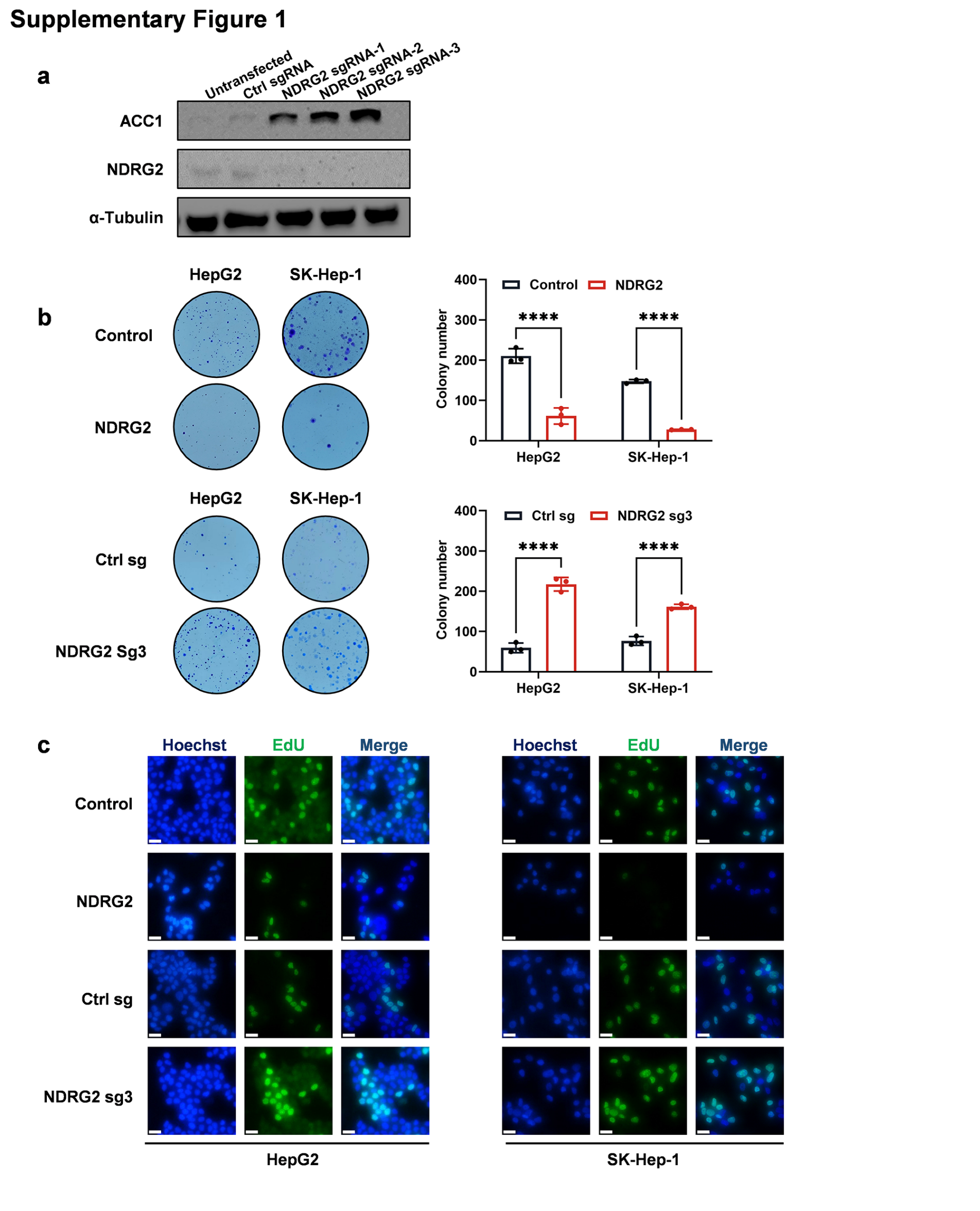
**Fig. S1: NDRG2 modulates the cell proliferation of hepatocellular carcinoma in vitro.** **a** Representative Western blot analysis showing protein levels of ACC1 and NDRG2 in cells subjected to different treatments. Cells were untransfected, transfected with Ctrl sgRNA, and transfected with three distinct sgRNAs targeting *NDRG2* (*NDRG2* sgRNA-1, -2, -3). α-Tubulin served as a loading control. **b** Colony formation assays were used to compare the ability of cell proliferation between *NDRG2*-overexpressing and *NDRG2*-knockout HCC cell lines HepG2 and SK-Hep-1 (n = 3 per group). The data are presented as means ± SEM (error bar) and compared using two-way ANOVA. *****P*<0.001. **c** EdU assay was used to analyze the effect of *NDRG2* overexpression and knockout on the cell proliferation of HCC cell lines HepG2 and SK-Hep-1 (Scale bars: 50 μm).

**
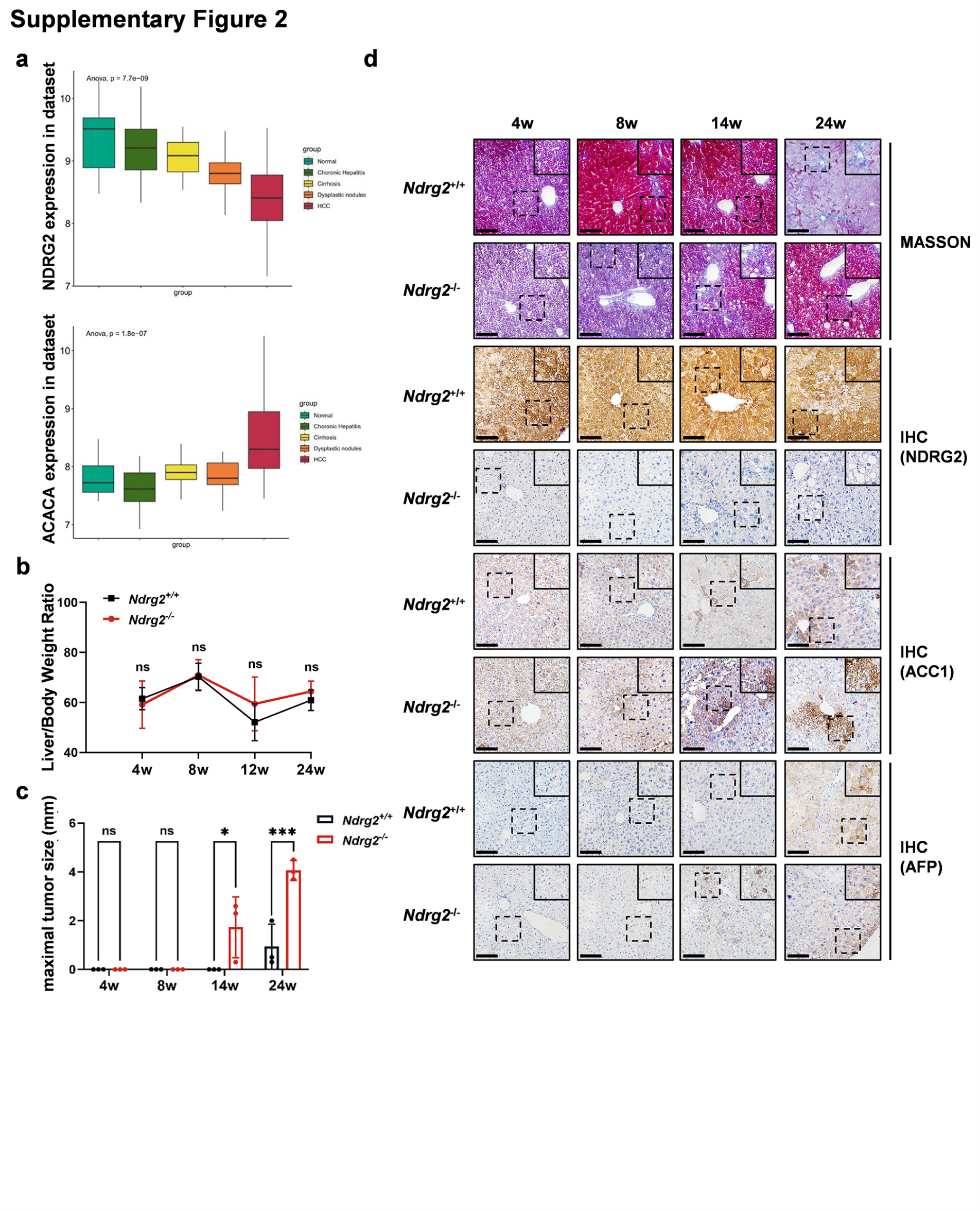
**

**Fig. S2: The *Ndrg2^-/-^* mouse model exhibits a heightened susceptibility to hepatocellular carcinoma induction.** **a** The expression of NDRG2 and ACC1 at the different stages of HCC development. **b** Quantification of the liver-to-body weight ratio in *Ndrg2^+/+^* and *Ndrg2^−/−^* mice (n = 6 per group). **c** Maximal tumor diameter of *Ndrg2^+/+^* and *Ndrg2^−/−^* mice (n = 3 per group). The data are presented as means ± SEM (error bar) and compared using two-way ANOVA. **P*=0.0497; ****P*=0.001. **d** Histopathological analysis of HCC tissues showing Masson (fibrosis), NDRG2 IHC (NDRG2 expression), ACC1 IHC (ACC1 expression), and AFP IHC (tumor marker) staining (Scale bars: 100 μm).

**
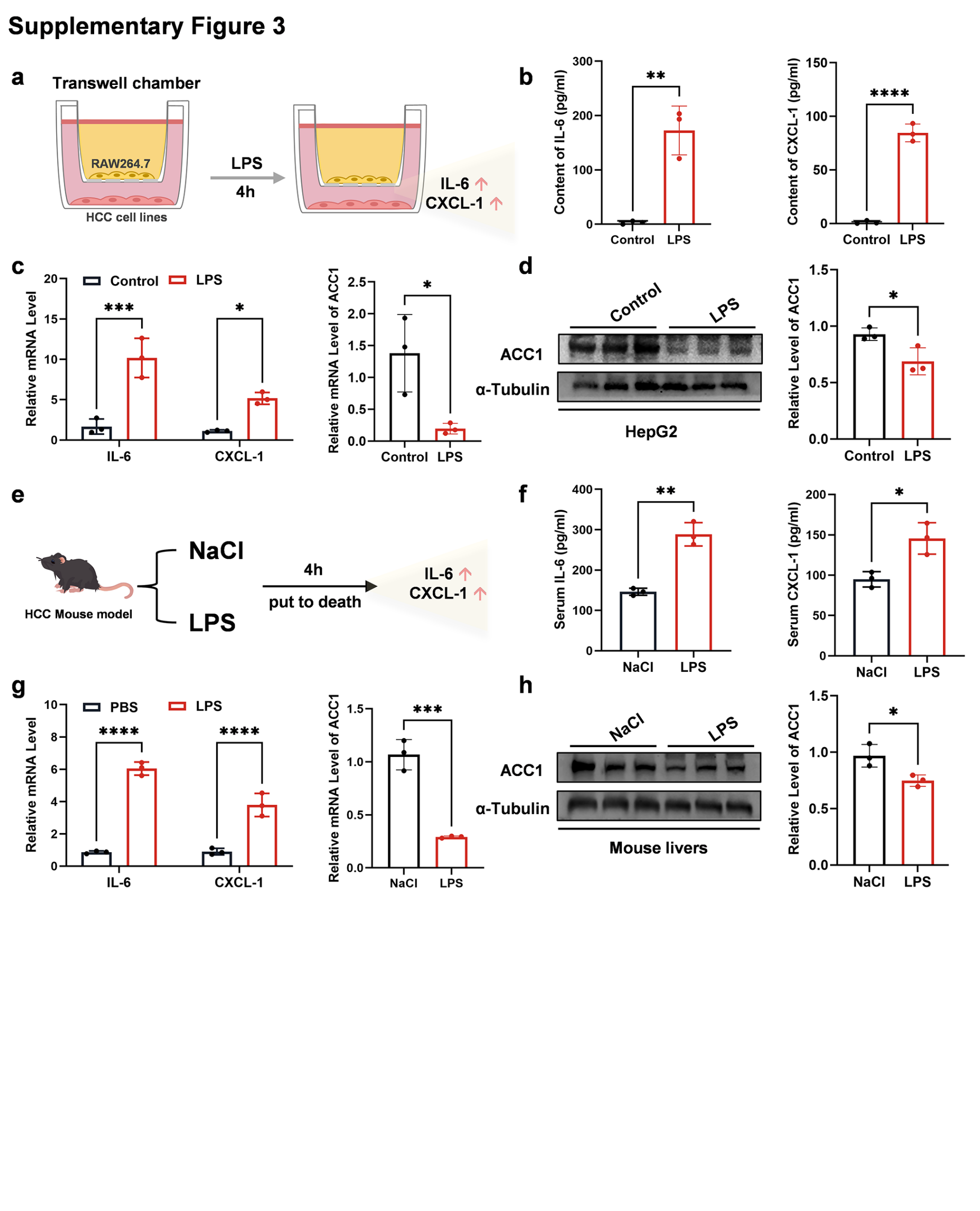
**

**Fig. S3:** **Short-term LPS stimulation downregulates ACC1 expression in hepatocellular carcinoma cells and a mouse HCC model. a** Schematic illustration of the Transwell co-culture system. RAW264.7 macrophages were seeded in the upper chamber and HCC cell lines in the lower chamber. This graphic was produced using PowerPoint software. **b** ELISA analysis showing the secretion levels of IL-6 and CXCL-1 in the culture medium of HepG2 cells after LPS stimulation (n = 3 per group). ***P*=0.0029. *****P*<0.0001**. c** RT-qPCR analysis of IL-6, CXCL-1 and ACC1 mRNA levels in HepG2 cells after LPS treatment (n=3 per group). ****P*=0.0001; **P*=0.0127; **P=*0.0286. **d** Western blot analysis of ACC1 protein level in HepG2 cells after LPS treatment. α-Tubulin served as a loading control (n=3 per group). **P*=0.0342. **e** Schematic diagram of the HCC mouse model. This graphic was produced using PowerPoint software. **f** ELISA analysis showing IL-6 and CXCL-1 protein levels in liver tissues of control and LPS-treated mice (n = 3 per group). ***P*=0.0012; **P*=0.0156. **g** RT-qPCR analysis of IL-6, CXCL-1 and ACC1 mRNA levels in liver tissues of control and LPS-treated mice (n = 3 per group). ****P* <0.001; *****P*<0.0001. **h** Western blot analysis of ACC1 protein level in liver tissues of control and LPS-treated mice. α-Tubulin served as a loading control (n = 3 per group). **P*=0.0274. **b-d** and **f-h** The data are presented as the means ± SEMs (error bars) and were compared using the two-way ANOVA and two-tailed unpaired t-test.

**
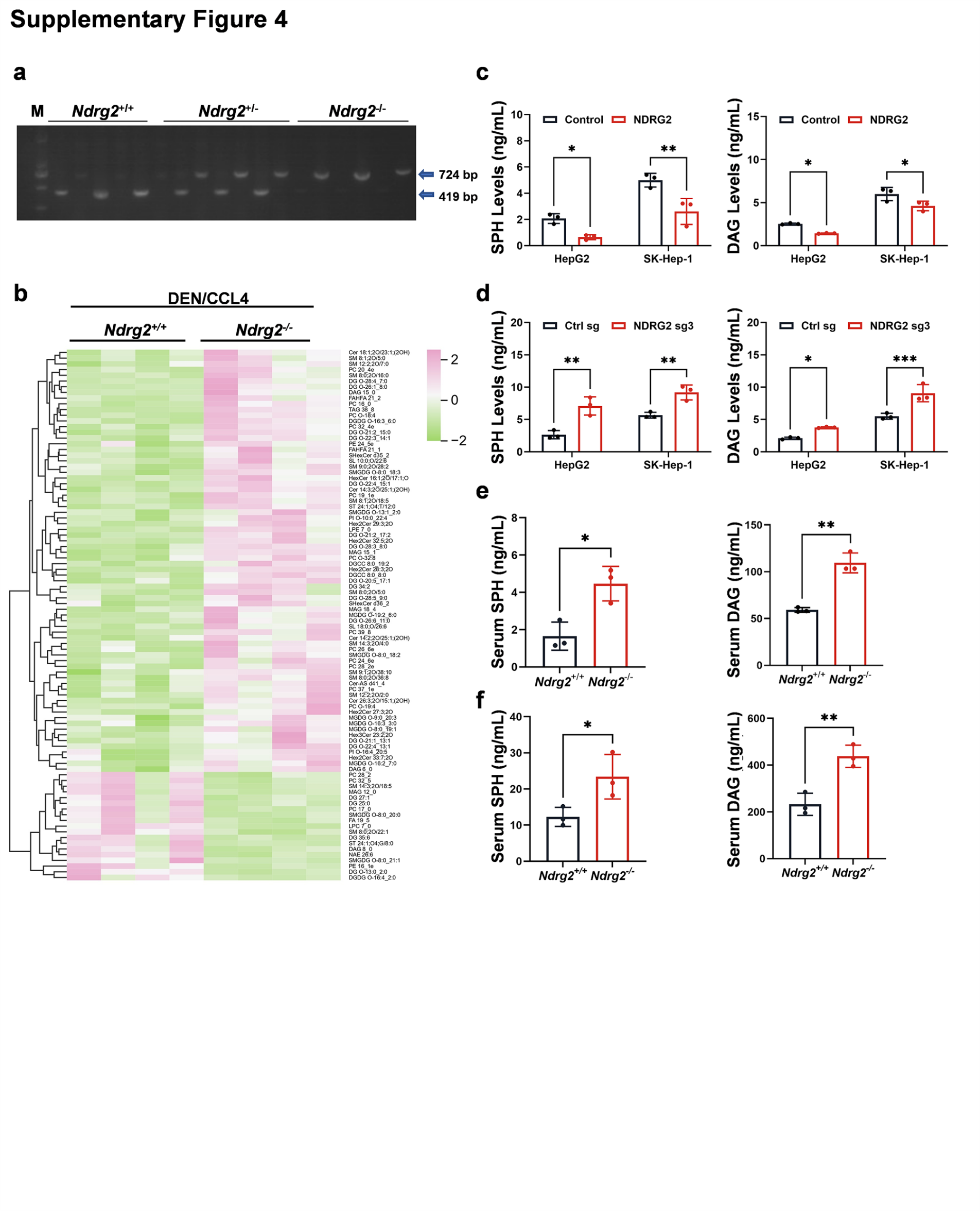
**

**Fig. S4: Lipidomic profiling of *NDRG2* knockout vs. control HepG2 cells, and *Ndrg2^+/+^* vs. *Ndrg2^-/-^* mouse tissues. a** Genotyping of mouse tail biopsies. **b** Lipidomic analysis reveals significant lipid differences in *Ndrg2^+/+^* vs. *Ndrg2^-/-^* mouse models induced by DEN/CCl_4_. **c** SPH (left) and DAG (right) in conditioned media from *NDRG2*-overexpressing and control HCC cells (n = 3 per group). **P*=0.0389; ***P*=0.0024; **P*=0.0426; **P*=0.0148. **d** SPH (left) and DAG (right) in conditioned media from *NDRG2*-knockout and control HCC cells (n = 3 per group). ***P*=0.0011; ***P*=0.0048; **P*=0.0409; ****P*=0.0005. **c-d** The data are presented as means ± SEM (error bar) and compared using two-way ANOVA. **e** SPH (left) and DAG (right) levels in serum of *Ndrg2^+/+^* and *Ndrg2^−/−^* mice (n = 3 per group). **P*=0.0150; ***P=*0.0013. **f** SPH (left) and DAG (right) levels in serum of *Ndrg2^+/+^* and *Ndrg2^-/-^* mice induced by DEN/CCl_4_ (n = 3 per group). **P*=0.032; ***P*=0.0097. **e-f** The data are presented as means ± SEM (error bar) and compared using two-tailed unpaired t-test.

**
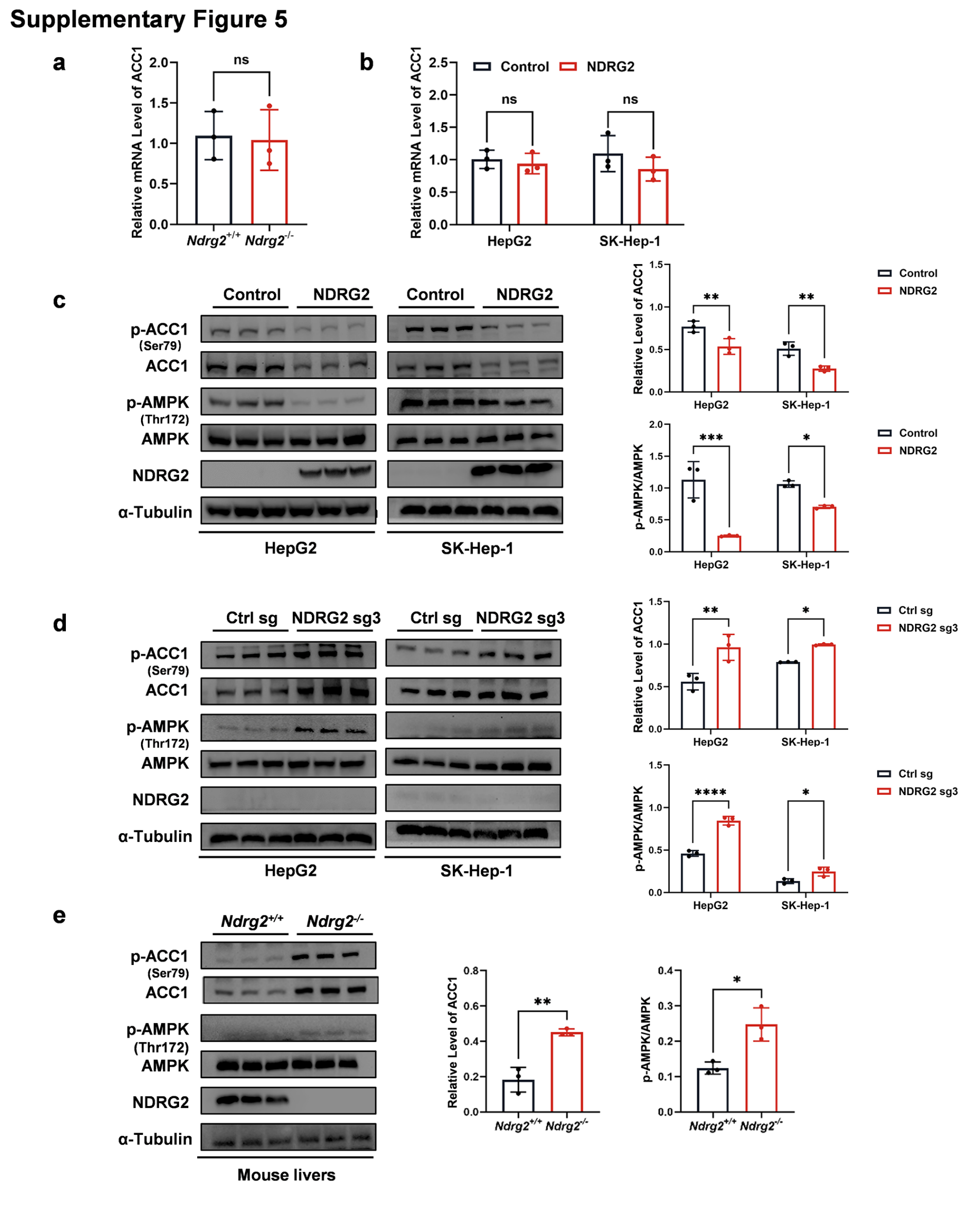
Fig. S5****: NDRG2 inhibits the phosphorylation of AMPK pathway. a** The mRNA level of ACC1 in liver tissues of *Ndrg2^+/+^* and *Ndrg2^-/-^* gene mice (n = 3 per group). The data are presented as means ± SEM (error bar) and compared using two-tailed unpaired t-test. **b** The mRNA levels of ACC1 in *NDRG2*-overexpressing HCC cell lines. **c-e** Representative Western blot analysis of p-ACC1 (Ser79), ACC1, p-AMPK (Thr172), AMPK and NDRG2 protein levels in the **(c)** *NDRG2*-overexpressing and control HCC cells (**d**) *NDRG2* knockout and control HCC cells and **(e)** *Ndrg2^+/+^* and *Ndrg2^-/-^* mouse liver tissues. α-Tubulin served as a loading control. **b-d** (n=3 per group). The data are presented as means ± SEM (error bar) and compared using two-way ANOVA. **P*<0.05; ***P*<0.01; ****P*<0.001. **e** The data are presented as means ± SEM (error bar) and compared using two-tailed unpaired t-test. ***P*=0.0032; **P*=0.0130.


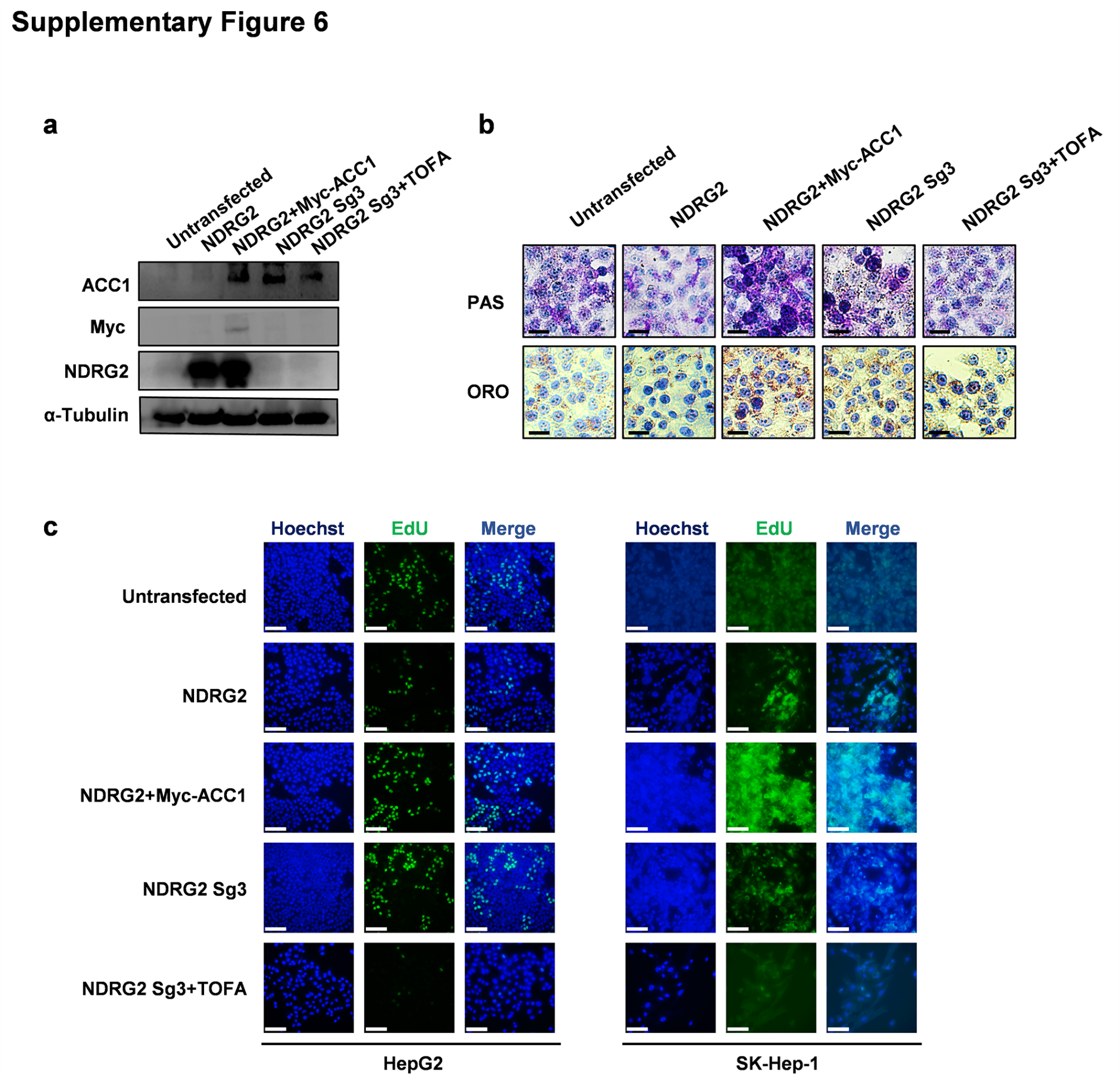


**Fig. S6: Genetic and functional rescue assays validate that NDRG2 acts as a negative regulator of ACC1-mediated lipogenesis. a** Western blot analysis of ACC1 protein level in HepG2 cells. **b** PAS and ORO staining results of HepG2 cells transfected with Myc-ACC1 or treated with TOFA (scale bars: 100 μm). **c** EdU staining results of HCC cells transfected with Myc-ACC1 or treated with TOFA (scale bars: 100 μm).

**
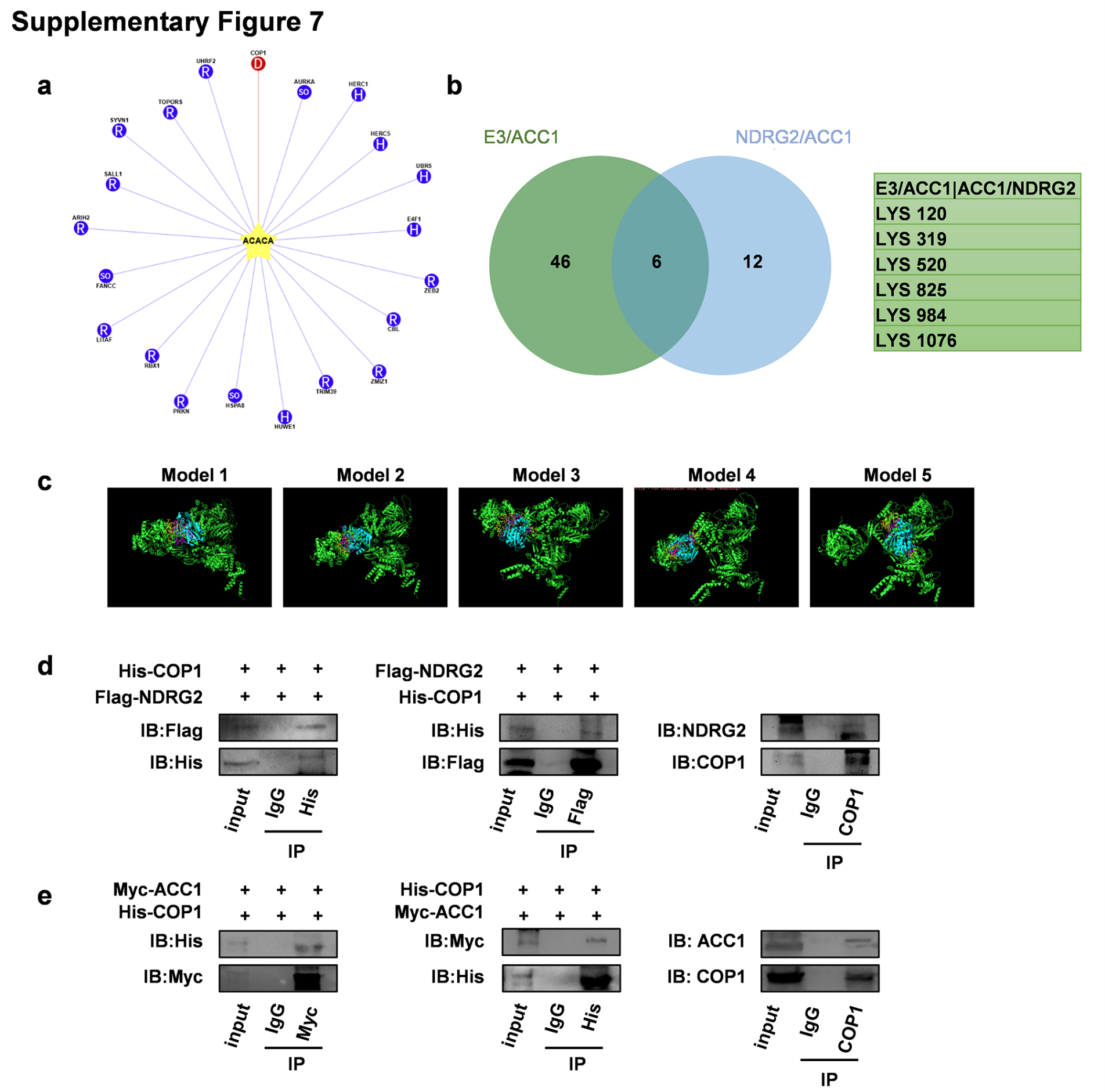
Fig. S7: Structural and biochemical validation of the NDRG2-COP1-ACC1 ternary complex formation. a** Analysis of E3 ubiquitin ligases that may bind to ACC1 using UbiBrowser 2.0. **b** Identifying the E3 ubiquitin ligase that interacts with both ACC1 and NDRG2. This graphic was produced using PowerPoint software. **c** Molecular docking simulation of NDRG2-ACC1 interaction. **d** Co-IP analysis of COP1-NDRG2 interaction in 293T cells and HepG2 cells. **e** Co-IP analysis of COP1-ACC1 interaction in 293T cells and HepG2 cells.


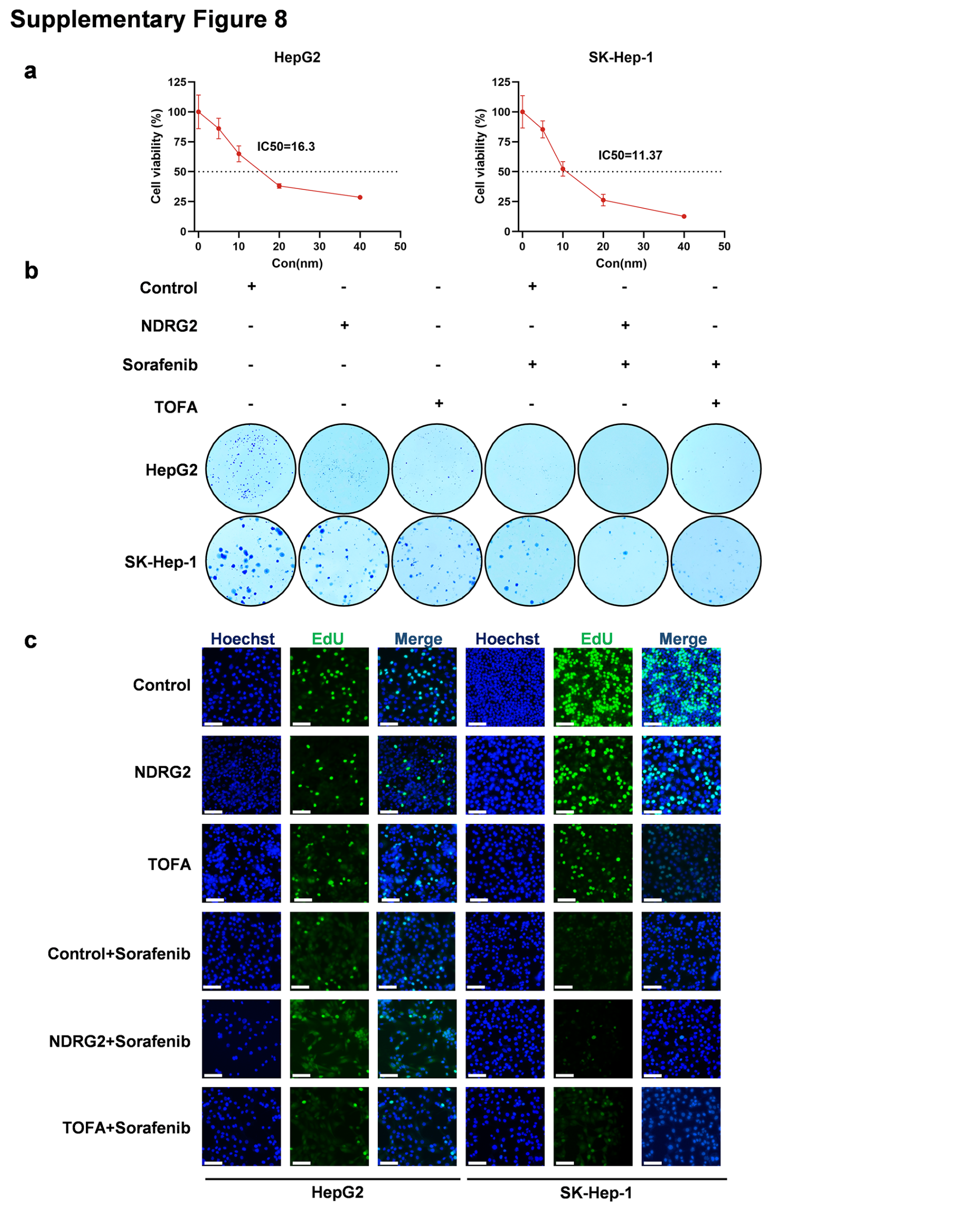
**Fig. S8: The effect of combining ACC1 and sorafenib in hepatocellular carcinoma cell lines.** **a** Cell viability of HCC cells after treatment with increasing concentrations of sorafenib for 24 hours, as measured by CCK‑8 assay. Data are presented as mean ± SD (n = 4 per group). The dotted line indicates 50% cell viability, and the arrow marks the corresponding (HepG2) IC₅₀ =16.3 nM; (SK-Hep-1) IC₅₀ =11.37 nM. **b** A colony-forming assay was performed to evaluate the effects of *NDRG2* overexpression and TOFA, alone or in combination with sorafenib, on the proliferation capacity of HCC cell lines. **c** EdU assay was used to analyze the effects of *NDRG2* overexpression and TOFA, alone or in combination with sorafenib, on cell proliferation capacity of HCC cell lines (scale bars: 100 μm).

**
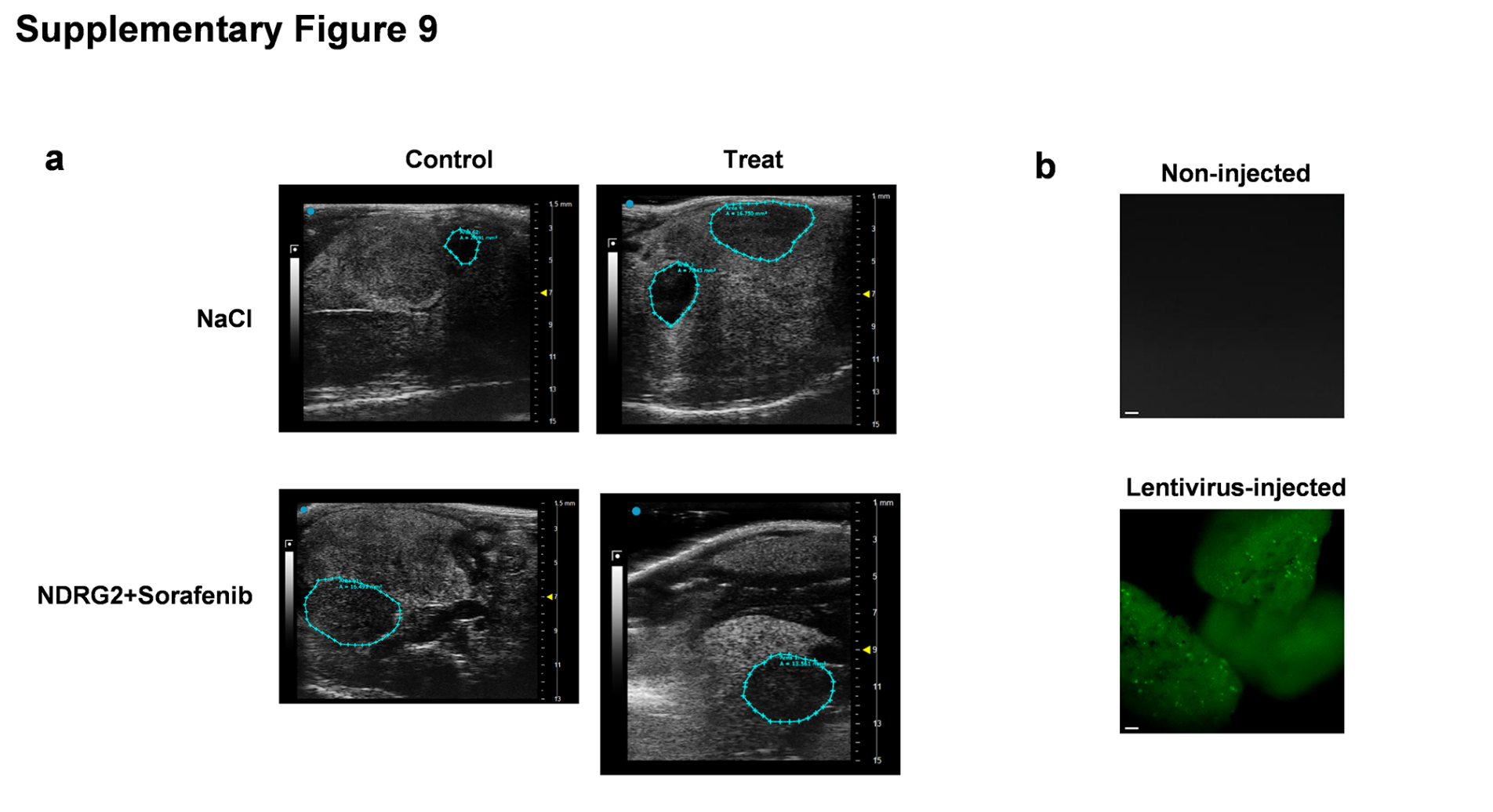
**

**Fig. S9: NDRG2-Sorafenib combination therapy demonstrates superior tumor regression in preclinical models. a** Comparison of liver ultrasound of HCC mice before and after NDRG2-Sorafenib combination treatment. **b** Fluorescence imaging of GFP-expressing liver four weeks after lentivirus contaning *NDRG2-EGFP* cDNA injection in the tail vein (Scale bar: 100 μm).

**
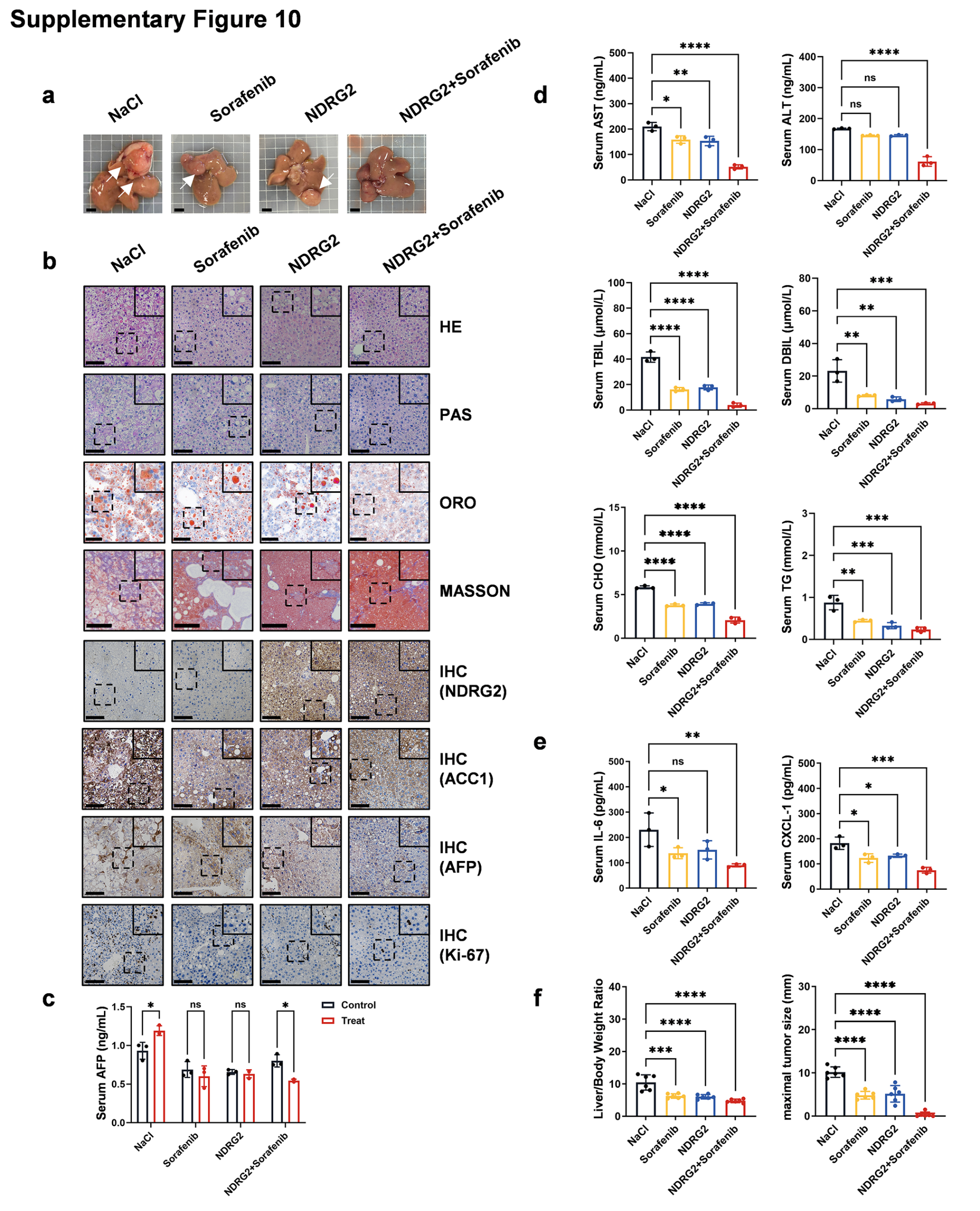
**

**Fig. S10: The effect of combining NDRG2 with sorafenib in female hepatocellular carcinoma mouse models. a** Representative gross liver morphology from the indicated treatment groups (Scale bar: 0.5 cm). **b** Histopathological analysis of HCC tissues from the indicated treatment groups showing H&E, PAS, ORO, Masson, NDRG2 IHC, ACC1 IHC, AFP IHC and Ki-67 IHC (Scale bars: 100 μm). **c** Serum AFP levels pre- and post-treatment (n = 3 per group). The data are presented as means ± SEM (error bar) and compared using two-way ANOVA. **P*=0.0237; **P*=0.027. **d** Serum levels of AST, ALT, TBIL, DBIL, CHO and TG in the female mice after the indicated treatment (n = 3 per group). **e** Serum cytokine levels of IL-6 and CXCL-1 (n = 3 per group). **f** Statistical analysis of liver-to-body weight ratio (left) and maximal tumor diameter (right) in female mice after the indicated treatment. (n = 6 per group). **d-f** The data are presented as means ± SEM (error bar) and compared using one-way ANOVA. **P*<0.05; ***P*<0.01; ****P*<0.001; *****P*<0.0001.


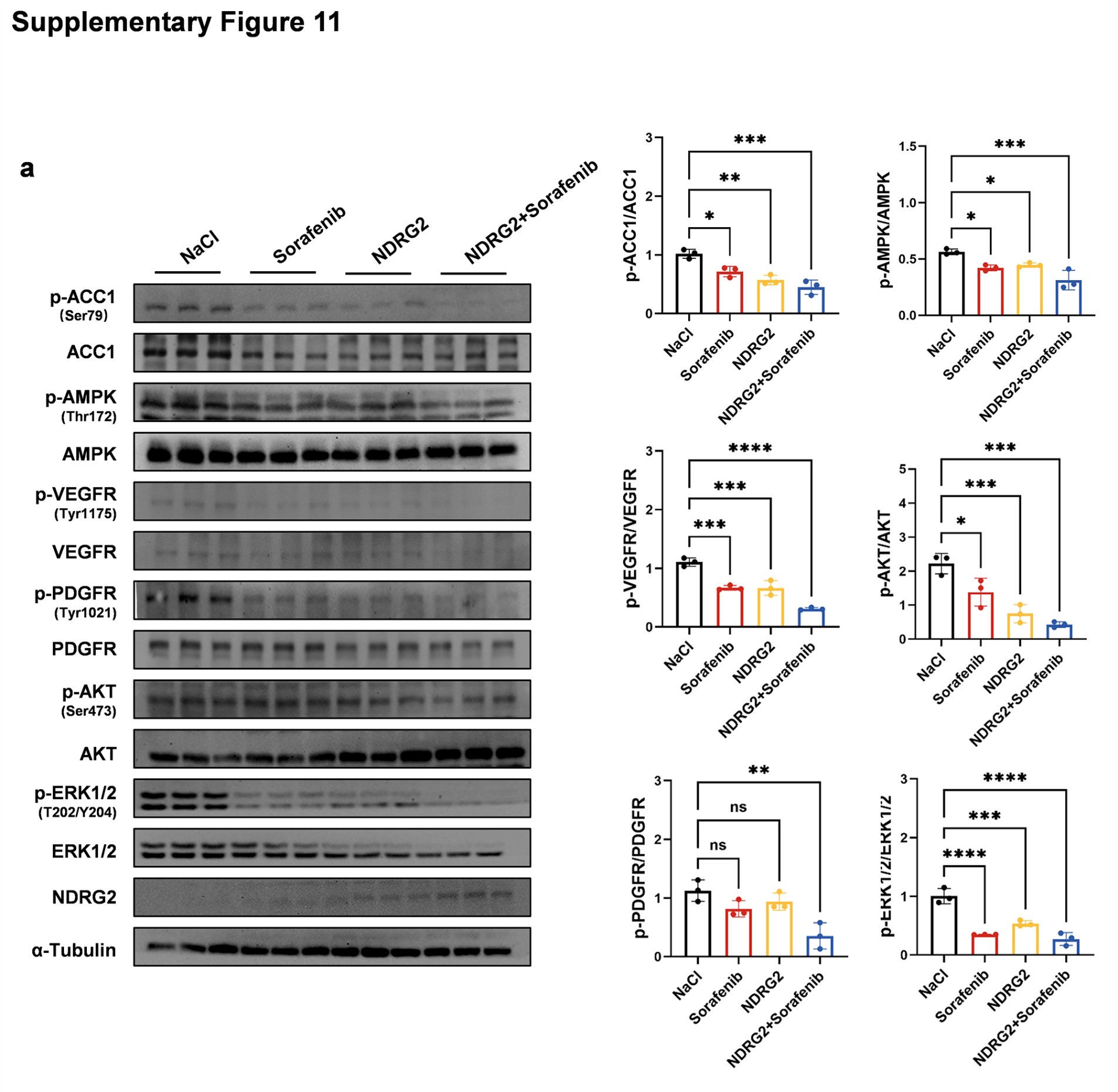


**Fig. S11:** **NDRG2 combined with Sorafenib affects ACC1 levels and angiogenesis pathways in hepatocellular carcinoma mouse models. a** Representative Western blot images showed the protein levels and phosphorylation states of key signaling molecules in mice subjected to the different treatments: NaCl, sorafenib treatment, NDRG2 overexpression, and the combination of NDRG2 overexpression with sorafenib. The blots were probed for p-ACC1 (Ser79), ACC1, p-AMPK (Thr172), AMPK, p-VEGFR (Tyr1175), VEGFR, p-PDGFR (Tyr1021), PDGFR, p-AKT (Ser473), AKT, p-ERK1/2 (T202/Y204), ERK1/2, and NDRG2. α-Tubulin served as the loading control (n = 3 per group). The data are presented as means ± SEM (error bar) and compared using one-way ANOVA. **P*<0.05; ***P*<0.01; ****P*<0.001; *****P*<0.0001.


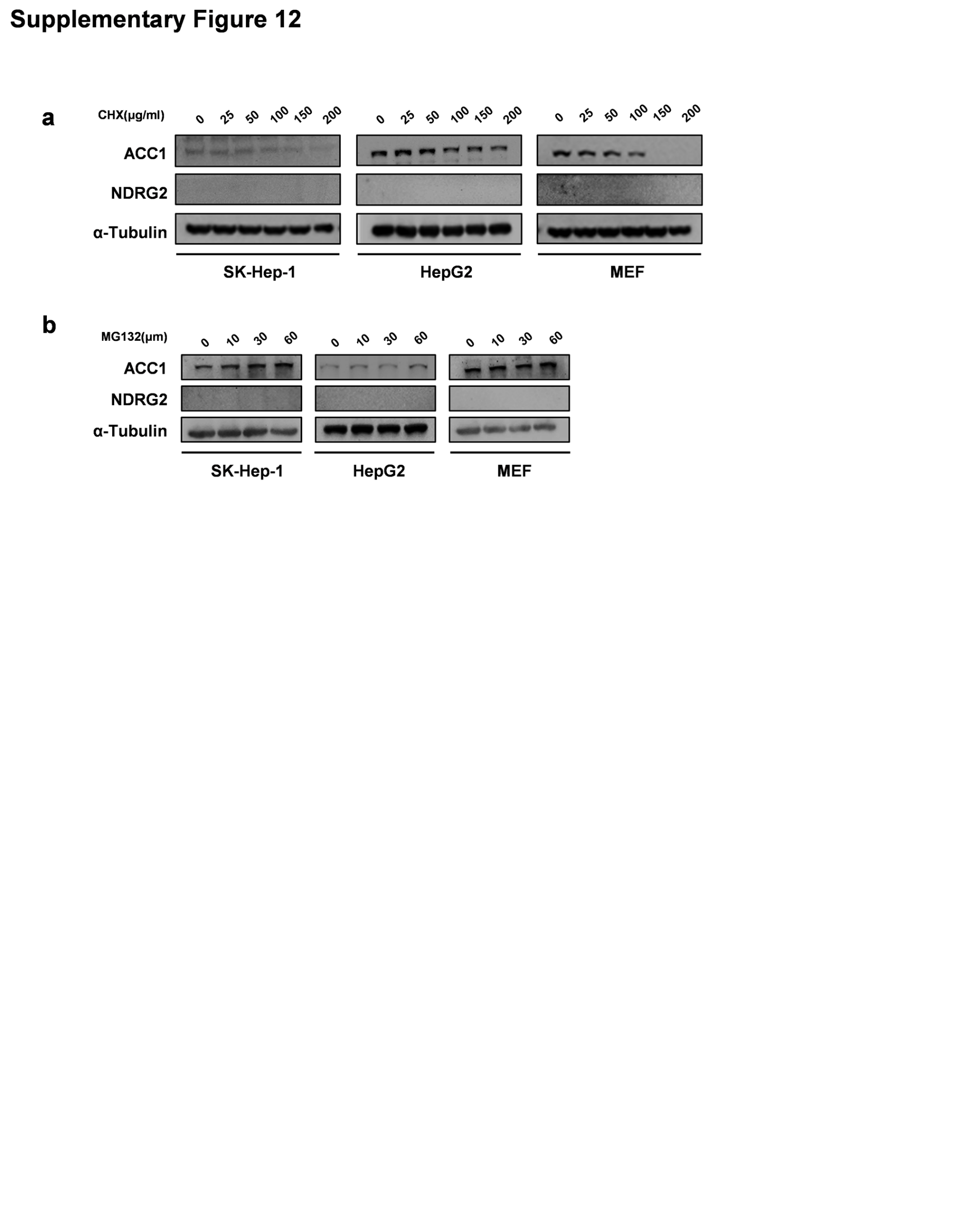


**Fig. S12: The effective working concentrations of Cycloheximide and MG132 were detected to analyze protein stability and degradation ways in HCC and MEF cells.**

**a** Western blot analysis of ACC1, NDRG2, and α-Tubulin protein levels in SK-Hep-1, HepG2 and MEF treated with CHX (0, 25, 50, 100, 150, 200 µg/ml) for 24 h. α-Tubulin served as a loading control. **b** Western blot analysis of ACC1, NDRG2, and α-Tubulin protein levels in SK-Hep-1, HepG2 and MEF treated with MG132 (0, 10, 30, 60 µM) for 6 h. α-Tubulin served as a loading control.
